# Supplementary material for: Polyketide synthase gene clusters in the ericoid mycorrhizal fungus Oidiodendron maius: genome-wide prediction, expression profiling, and functional characterization of knockout mutants
Source: Front Plant Sci. 2026 Sep 16;17:1924430. doi: 10.3389/fpls.2026.1924430 (PMC13623903; doi:10.3389/fpls.2026.1924430)
Supplement: Supplementary file 1 [file SupplementaryFile1.zip › Supplementary Materials/Supplementary Folder 1.zip!/Oidma1_AssemblyScaffolds_Repeatmasked/knownclusterblast/cluster4/jgi.p_Oidma1_111074_mibig_hits.html]

| MIBiG Protein | Description | MIBiG Cluster | MiBiG Product | % ID | % Coverage | BLAST Score | E-value |
| --- | --- | --- | --- | --- | --- | --- | --- |
| EAU38791.1 | hypothetical\_protein | BGC0000161 | Terrein\_biosynthetic\_gene\_cluster | 60 | 100.952834683 | 2471 | 0.0 |
| BAD22832.1 | polyketide\_synthase | BGC0001265 | Melanin\_biosynthetic\_gene\_cluster | 48 | 97.5226298237 | 1836 | 0.0 |
| AAN59953.1 | polyketide\_synthase\_1 | BGC0001258 | 1,8-dihydroxynaphthalene\_biosynthetic\_gene\_cluster | 47 | 97.2367794188 | 1762 | 0.0 |
| AAD38786.1 | polyketide\_synthase | BGC0001257 | 1,3,6,8-tetrahydroxynaphthalene\_biosynthetic\_gene\_cluster | 45 | 97.5226298237 | 1699 | 0.0 |
| CBF74114.1 | Conidial\_yellow\_pigment\_biosynthesis\_polyketide\_synthase\_(PKS)(EC\_2.3.1.-)\_[Source:UniProtKB/Swiss-Prot;Acc:Q03149] | BGC0000107 | Naphthopyrone\_biosynthetic\_gene\_cluster | 45 | 98.0466888995 | 1680 | 0.0 |
| ADM79459.1 | PKS16\_protein | BGC0001266 | Grayanic\_acid\_biosynthetic\_gene\_cluster | 41 | 97.1891376846 | 1456 | 0.0 |
| ABU63483.1 | polyketide\_synthase | BGC0001259 | Elsinochrome\_A\_biosynthetic\_gene\_cluster | 47 | 72.9871367318 | 1418 | 0.0 |
| EAL89339.1 | polyketide\_synthase,\_putative | BGC0001403 | Trypacidin\_biosynthetic\_gene\_cluster | 44 | 78.0371605526 | 1291 | 0.0 |
| CBF90097.1 | hypothetical\_protein\_similar\_to\_polyketide\_synthase\_(Broad) | BGC0001271 | Emericellin\_biosynthetic\_gene\_cluster | 43 | 78.2753692234 | 1288 | 0.0 |
| AGO59040.1 | PtaA | BGC0000121 | Pestheic\_acid\_biosynthetic\_gene\_cluster | 43 | 78.8470700333 | 1270 | 0.0 |
| AAT69682.1 | polyketide\_synthase | BGC0001247 | Cercosporin\_biosynthetic\_gene\_cluster | 36 | 99.9523582658 | 1239 | 0.0 |
| ACH72912.1 | AflC | BGC0000011 | Aflatoxin/sterigmatocystin\_biosynthetic\_gene\_cluster | 36 | 100.57170081 | 1223 | 0.0 |
| CCT67991.1 | bikaverin\_cluster-polyketide\_synthase | BGC0000030 | Bikaverin\_biosynthetic\_gene\_cluster | 41 | 79.5140543116 | 1215 | 0.0 |
| CCE67070.1 | polyketide\_synthase | BGC0001242 | Fusarubin\_biosynthetic\_gene\_cluster | 40 | 82.5154835636 | 1210 | 0.0 |
| CCH26295.1 | putative\_polyketide\_synthase | BGC0001158\_c6 | Bikaverin\_biosynthetic\_gene\_cluster | 40 | 84.3735111958 | 1203 | 0.0 |
| AAC49191.1 | putative\_polyketide\_synthase | BGC0000152 | Sterigmatocystin\_biosynthetic\_gene\_cluster | 35 | 101.048118152 | 1177 | 0.0 |
| EGD99348.1 | polyketide\_synthase | BGC0001144 | Neosartoricin\_biosynthetic\_gene\_cluster | 40 | 73.939971415 | 1151 | 0.0 |
| EAL84397.1 | polyketide\_synthase,\_putative | BGC0001118 | Endocrocin\_biosynthetic\_gene\_cluster | 42 | 71.9390185803 | 1146 | 0.0 |
| CBF70387.1 | polyketide\_synthase,\_putative\_(JCVI) | BGC0000684 | Asperthecin\_biosynthetic\_gene\_cluster | 41 | 73.0347784659 | 1138 | 0.0 |
| AKN45693.1 | polyketide\_synthase | BGC0001284 | Alternariol\_biosynthetic\_gene\_cluster | 40 | 76.9890424011 | 1107 | 0.0 |
| AEN83889.1 | AdaA | BGC0000156 | TAN-1612\_biosynthetic\_gene\_cluster | 39 | 73.8446879466 | 1092 | 0.0 |
| ADI24926.1 | VrtA | BGC0000168 | Viridicatumtoxin\_biosynthetic\_gene\_cluster | 39 | 73.3206288709 | 1074 | 1.5e-313 |
| AAS89999.1 | PksA | BGC0000007 | Aflatoxin\_biosynthetic\_gene\_cluster | 35 | 87.9466412577 | 1068 | 1.1e-311 |
| AAS90093.1 | PksA | BGC0000006 | Aflatoxin\_biosynthetic\_gene\_cluster | 36 | 86.3744640305 | 1055 | 5.8e-308 |
| ADI24953.1 | GsfA | BGC0000070 | Griseofulvin\_biosynthetic\_gene\_cluster | 40 | 72.0819437828 | 1052 | 6.4e-307 |
| EED53479.1 | polyketide\_synthase,\_putative | BGC0001304 | Aflavarin\_biosynthetic\_gene\_cluster | 38 | 78.2277274893 | 1048 | 9.3e-306 |
| BAE71314.1 | polyketide\_synthase | BGC0000004 | Aflatoxin\_biosynthetic\_gene\_cluster | 37 | 81.6579323487 | 1045 | 1e-304 |
| AAS90047.1 | PksA | BGC0000009 | Aflatoxin\_biosynthetic\_gene\_cluster | 36 | 86.2791805622 | 1039 | 4.3e-303 |
| AAS90022.1 | PksA | BGC0000008 | Aflatoxin\_biosynthetic\_gene\_cluster | 36 | 80.1810385898 | 1026 | 3.8e-299 |
| CBF79143.1 | polyketide\_synthase,\_putative\_(JCVI) | BGC0000013 | Alternariol\_biosynthetic\_gene\_cluster | 38 | 75.0833730348 | 967 | 3.6e-281 |
| EAA59563.1 | hypothetical\_protein | BGC0000057 | F9775\_biosynthetic\_gene\_cluster | 32 | 99.9047165317 | 927 | 2.4e-269 |
| AAZ95017.1 | polyketide\_synthase | BGC0000048 | Dothistromin\_biosynthetic\_gene\_cluster | 42 | 54.9785612196 | 887 | 3.6e-257 |
| AGC95321.1 | CurS2 | BGC0000045 | Dehydrocurvularin\_biosynthetic\_gene\_cluster | 35 | 75.3692234397 | 873 | 5.4e-253 |
| ABB90282.1 | polyketide\_synthase | BGC0001057 | Zearalenone\_biosynthetic\_gene\_cluster | 34 | 76.655550262 | 865 | 1.5e-250 |
| ACD39762.1 | non-reducing\_polyketide\_synthase | BGC0000077 | Hypothemycin\_biosynthetic\_gene\_cluster | 33 | 78.2753692234 | 852 | 7.5e-247 |
| ACD39753.1 | non-reducing\_polyketide\_synthase | BGC0000076 | Hypothemycin\_biosynthetic\_gene\_cluster | 33 | 78.2753692234 | 852 | 9.8e-247 |
| AHV78247.1 | LasS2 | BGC0001245 | Lasiodiplodin\_biosynthetic\_gene\_cluster | 32 | 80.1810385898 | 841 | 2.3e-243 |
| AHV78253.1 | ResS2 | BGC0001246 | Trans-resorcylide\_biosynthetic\_gene\_cluster | 33 | 82.8966174369 | 837 | 2.5e-242 |
| AAS92537.1 | PKS1 | BGC0001044 | Sirodesmin\_biosynthetic\_gene\_cluster | 41 | 54.6927108147 | 836 | 9.5e-242 |
| ACD39770.1 | non-reducing\_polyketide\_synthase | BGC0000134 | Radicicol\_biosynthetic\_gene\_cluster | 31 | 77.5131014769 | 777 | 5.2e-224 |
| AAK57187.1 | MxaC | BGC0001022 | Myxalamid\_biosynthetic\_gene\_cluster | 35 | 43.3063363506 | 535 | 3.5e-151 |
| BAV19379.1 | polyketide\_synthase | BGC0001390 | LL-Z1272beta\_biosynthetic\_gene\_cluster | 27 | 83.5159599809 | 521 | 5.2e-147 |
| AGC45620.1 | polyketide\_synthase | BGC0001394 | Phenalamide\_biosynthetic\_gene\_cluster | 30 | 59.9333015722 | 517 | 5.7e-146 |
| AFV96138.1 | polyketide\_synthase | BGC0001064 | Cylindrocyclophane\_biosynthetic\_gene\_cluster | 36 | 42.8299190091 | 515 | 2.2e-145 |
| ADY00130.1 | polyketide\_synthase | BGC0000104 | Mycophenolic\_acid\_biosynthetic\_gene\_cluster | 29 | 68.9375893283 | 514 | 8.3e-145 |
| AIW82279.1 | PuwB | BGC0001125 | Puwainaphycins\_biosynthetic\_gene\_cluster | 33 | 51.5483563602 | 512 | 2.4e-144 |
| ADF88276.1 | polyketide\_synthase | BGC0000981 | Cylindrospermopsin\_biosynthetic\_gene\_cluster | 33 | 47.7846593616 | 510 | 7e-144 |
| AAS98783.1 | JamL | BGC0001001 | Jamaicamide\_biosynthetic\_gene\_cluster | 34 | 43.9733206289 | 510 | 1.2e-143 |
| CAD89777.1 | MelF\_protein | BGC0001009 | Melithiazol\_biosynthetic\_gene\_cluster | 34 | 43.0681276798 | 509 | 1.6e-143 |
| AAW03329.1 | CtaF | BGC0000982 | Cystothiazole\_A\_biosynthetic\_gene\_cluster | 34 | 42.9728442115 | 508 | 2.7e-143 |
| ABX60152.1 | polyketide\_synthase | BGC0000978 | Cylindrospermopsin\_biosynthetic\_gene\_cluster | 33 | 47.7846593616 | 505 | 2.9e-142 |
| ABM21570.1 | CrpB | BGC0000975 | Cryptophycin\_biosynthetic\_gene\_cluster | 34 | 43.6398284898 | 504 | 6.5e-142 |
| AAF19814.1 | MtaF | BGC0001024 | Myxothiazol\_biosynthetic\_gene\_cluster | 33 | 43.401619819 | 502 | 3.2e-141 |
| AAW03328.1 | CtaE | BGC0000982 | Cystothiazole\_A\_biosynthetic\_gene\_cluster | 34 | 44.1638875655 | 499 | 2.1e-140 |
| CAD19093.1 | StiJ\_protein | BGC0000153 | Stigmatellin\_biosynthetic\_gene\_cluster | 35 | 43.115769414 | 497 | 1e-139 |
| CAQ18829.1 | polyketide\_synthase | BGC0000954 | Ajudazol\_biosynthetic\_gene\_cluster | 34 | 43.6874702239 | 497 | 1e-139 |
| CAQ18834.1 | polyketide\_synthase | BGC0000954 | Ajudazol\_biosynthetic\_gene\_cluster | 36 | 42.7346355407 | 496 | 1.4e-139 |
| CAD19086.1 | StiB\_protein | BGC0000153 | Stigmatellin\_biosynthetic\_gene\_cluster | 35 | 43.1634111482 | 495 | 4e-139 |
| AEU11006.1 | NpnB | BGC0001029 | Nostophycin\_biosynthetic\_gene\_cluster | 33 | 44.6879466413 | 493 | 1.2e-138 |
| AAF19813.1 | MtaE | BGC0001024 | Myxothiazol\_biosynthetic\_gene\_cluster | 34 | 43.5445450214 | 492 | 2.6e-138 |
| CBD77748.1 | polyketide\_synthase | BGC0000974 | Crocacin\_biosynthetic\_gene\_cluster | 33 | 47.9275845641 | 490 | 7.5e-138 |
| AEE88289.1 | CurA | BGC0000976 | Curacin\_biosynthetic\_gene\_cluster | 33 | 45.7837065269 | 490 | 9.8e-138 |
| AAT70096.1 | CurA | BGC0001165 | Curacin\_biosynthetic\_gene\_cluster | 33 | 45.7837065269 | 490 | 9.8e-138 |
| AAT70103.1 | CurH | BGC0001165 | Curacin\_biosynthetic\_gene\_cluster | 33 | 43.0204859457 | 488 | 4.9e-137 |
| AEE88282.1 | CurH | BGC0000976 | Curacin\_biosynthetic\_gene\_cluster | 33 | 43.0204859457 | 488 | 4.9e-137 |
| AAT70108.1 | CurM | BGC0001165 | Curacin\_biosynthetic\_gene\_cluster | 34 | 43.5921867556 | 487 | 1.1e-136 |
| AEE88277.1 | CurM | BGC0000976 | Curacin\_biosynthetic\_gene\_cluster | 34 | 43.5921867556 | 487 | 1.1e-136 |
| ACV42478.1 | polyketide\_synthase | BGC0000043 | Curacin\_A\_biosynthetic\_gene\_cluster | 34 | 43.5921867556 | 487 | 1.1e-136 |
| CDM36726.1 | Beta-ketoacyl\_synthase | BGC0001360 | Mycophenolic\_acid\_biosynthetic\_gene\_cluster | 28 | 65.9361600762 | 486 | 1.8e-136 |
| AEE88279.1 | CurK | BGC0000976 | Curacin\_biosynthetic\_gene\_cluster | 32 | 50.9766555503 | 485 | 4.1e-136 |
| AAT70106.1 | CurK | BGC0001165 | Curacin\_biosynthetic\_gene\_cluster | 32 | 50.9766555503 | 485 | 4.1e-136 |
| CAD19087.1 | StiC\_protein | BGC0000153 | Stigmatellin\_biosynthetic\_gene\_cluster | 34 | 47.6893758933 | 485 | 4.1e-136 |
| AIW82282.1 | PuwE | BGC0001125 | Puwainaphycins\_biosynthetic\_gene\_cluster | 34 | 43.3063363506 | 484 | 7e-136 |
| CCE88376.1 | polyketide\_synthase | BGC0001034 | Pellasoren\_biosynthetic\_gene\_cluster | 33 | 47.9752262982 | 483 | 1.2e-135 |
| XP\_011392701.1 | hypothetical\_protein | BGC0001281 | Ustilagic\_acid\_biosynthetic\_gene\_cluster | 32 | 45.212005717 | 483 | 1.6e-135 |
| AAU04878.1 | polyketide\_synthase | BGC0000365 | Glycopeptidolipid\_biosynthetic\_gene\_cluster | 32 | 48.8804192473 | 481 | 5.9e-135 |
| ABX60163.1 | polyketide\_synthase | BGC0000978 | Cylindrospermopsin\_biosynthetic\_gene\_cluster | 33 | 44.0686040972 | 480 | 1.3e-134 |
| ADF88277.1 | polyketide\_synthase | BGC0000981 | Cylindrospermopsin\_biosynthetic\_gene\_cluster | 32 | 46.7841829443 | 479 | 1.7e-134 |
| AGN71604.1 | conidial\_yellow\_pigment\_biosynthesis\_polyketide\_synthase | BGC0000027 | Azaphilone\_biosynthetic\_gene\_cluster | 26 | 87.9942829919 | 478 | 3.8e-134 |
| CAQ18833.1 | polyketide\_synthase | BGC0000954 | Ajudazol\_biosynthetic\_gene\_cluster | 34 | 44.8308718437 | 477 | 8.6e-134 |
| AHH34186.1 | polyketide\_synthase | BGC0001161 | 1-heptadecene\_biosynthetic\_gene\_cluster | 32 | 45.212005717 | 476 | 1.5e-133 |
| CBD77736.1 | polyketide\_synthase | BGC0000974 | Crocacin\_biosynthetic\_gene\_cluster | 34 | 48.0705097666 | 476 | 1.9e-133 |
| CAQ34920.1 | polyketide\_synthase | BGC0000986 | Dkxanthene\_biosynthetic\_gene\_cluster | 33 | 48.4040019057 | 475 | 2.5e-133 |
| AAK57189.1 | MxaE | BGC0001022 | Myxalamid\_biosynthetic\_gene\_cluster | 32 | 47.9752262982 | 474 | 5.5e-133 |
| CAO98849.1 | polyketide\_synthase\_AufF | BGC0000023 | Aurafuron\_biosynthetic\_gene\_cluster | 32 | 51.500714626 | 474 | 7.2e-133 |
| AHB82064.1 | polyketide\_synthase | BGC0001231 | Microsclerodermins\_biosynthetic\_gene\_cluster | 35 | 43.7827536922 | 472 | 2.1e-132 |
| CAD89776.1 | MelE\_protein | BGC0001010 | Melithiazol\_biosynthetic\_gene\_cluster | 35 | 43.3539780848 | 470 | 1.4e-131 |
| AAF62883.1 | epoD | BGC0000991 | Epothilone\_biosynthetic\_gene\_cluster | 33 | 46.9271081467 | 470 | 1.4e-131 |
| ADB12491.1 | EpoD | BGC0000990 | Epothilone\_biosynthetic\_gene\_cluster | 33 | 46.9271081467 | 469 | 2.3e-131 |
| AID65222.1 | putative\_aspartate\_racemase | BGC0000335 | Cystomanamides\_biosynthetic\_gene\_cluster | 33 | 44.4020962363 | 469 | 2.3e-131 |
| ALI92655.1 | CitS\_citrinin\_polyketide\_synthase | BGC0001338 | Citrinin\_biosynthetic\_gene\_cluster | 27 | 74.6069556932 | 468 | 4e-131 |
| ACB46195.1 | polyketide\_synthase | BGC0000989 | Epothilone\_biosynthetic\_gene\_cluster | 33 | 46.9271081467 | 467 | 6.8e-131 |
| AHA12079.1 | polyketide\_synthase\_type\_1 | BGC0001172 | Chlorizidine\_A\_biosynthetic\_gene\_cluster | 34 | 44.2591710338 | 467 | 1.2e-130 |
| AAF00959.1 | McyD | BGC0001017 | Microcystin\_biosynthetic\_gene\_cluster | 34 | 44.8308718437 | 465 | 2.6e-130 |
| AAF26921.1 | polyketide\_synthase | BGC0000988 | Epothilone\_biosynthetic\_gene\_cluster | 32 | 46.9271081467 | 465 | 2.6e-130 |
| ADZ24998.1 | polyketide\_synthase | BGC0000380 | Leupyrrin\_biosynthetic\_gene\_cluster | 34 | 43.3539780848 | 465 | 4.4e-130 |
| CAQ34919.1 | polyketide\_synthase | BGC0000986 | Dkxanthene\_biosynthetic\_gene\_cluster | 31 | 51.0242972844 | 464 | 7.5e-130 |
| AFU82617.1 | polyketide\_synthase | BGC0000998 | Guadinomine\_biosynthetic\_gene\_cluster | 34 | 44.8308718437 | 463 | 9.8e-130 |
| ADN13832.1 | Beta-ketoacyl\_synthase | BGC0001164 | 1-heptadecene\_biosynthetic\_gene\_cluster | 33 | 43.4492615531 | 463 | 1.3e-129 |
| AAO62584.1 | polyketide\_synthase\_type\_1 | BGC0001016 | Microcystin\_biosynthetic\_gene\_cluster | 34 | 43.115769414 | 462 | 2.2e-129 |
| CAD19090.1 | StiF\_protein | BGC0000153 | Stigmatellin\_biosynthetic\_gene\_cluster | 33 | 48.4516436398 | 462 | 2.9e-129 |
| ACR33079.1 | polyketide\_synthase | BGC0000017 | Anatoxin\_biosynthetic\_gene\_cluster | 33 | 43.4969032873 | 460 | 1.1e-128 |
| AAS98782.1 | JamK | BGC0001001 | Jamaicamide\_biosynthetic\_gene\_cluster | 34 | 42.3535016675 | 460 | 1.1e-128 |
| CBF80428.1 | polyketide\_synthase,\_putative\_(JCVI) | BGC0000680 | Austinol/dehydroaustinol,\_locus\_1\_biosynthetic\_gene\_cluster | 28 | 68.556455455 | 460 | 1.1e-128 |
| AFU82616.1 | polyketide\_synthase | BGC0000998 | Guadinomine\_biosynthetic\_gene\_cluster | 34 | 43.0204859457 | 459 | 2.4e-128 |
| CAD19089.1 | StiE\_protein | BGC0000153 | Stigmatellin\_biosynthetic\_gene\_cluster | 32 | 42.9728442115 | 456 | 1.2e-127 |
| CCE88379.1 | polyketide\_synthase | BGC0001034 | Pellasoren\_biosynthetic\_gene\_cluster | 32 | 48.2610767032 | 456 | 1.2e-127 |
| AHB82053.1 | polyketide\_synthase | BGC0001019 | Microsclerodermins\_biosynthetic\_gene\_cluster | 34 | 43.115769414 | 455 | 2.7e-127 |
| CAO98847.1 | polyketide\_synthase\_AufC | BGC0000023 | Aurafuron\_biosynthetic\_gene\_cluster | 33 | 42.9252024774 | 455 | 2.7e-127 |
| AGC45621.1 | polyketide\_synthase | BGC0001394 | Phenalamide\_biosynthetic\_gene\_cluster | 32 | 48.5469271081 | 455 | 3.5e-127 |
| CAL58685.1 | polyketide\_synthase | BGC0000149 | Spirangien\_biosynthetic\_gene\_cluster | 32 | 48.499285374 | 454 | 5.9e-127 |
| AGC45623.1 | polyketide\_synthase | BGC0001394 | Phenalamide\_biosynthetic\_gene\_cluster | 35 | 43.5921867556 | 453 | 1.3e-126 |
| ADZ24996.1 | polyketide\_synthase | BGC0000380 | Leupyrrin\_biosynthetic\_gene\_cluster | 32 | 48.1181515007 | 453 | 1.3e-126 |
| AHA12078.1 | polyketide\_synthase\_type\_1 | BGC0001172 | Chlorizidine\_A\_biosynthetic\_gene\_cluster | 33 | 44.2115292997 | 453 | 1.7e-126 |
| ADZ24997.1 | polyketide\_synthase | BGC0000380 | Leupyrrin\_biosynthetic\_gene\_cluster | 32 | 47.6417341591 | 452 | 2.9e-126 |
| AFP87523.1 | type\_I\_polyketide\_synthase | BGC0001159 | Marinopyrrole\_biosynthetic\_gene\_cluster | 32 | 43.2110528823 | 451 | 3.9e-126 |
| AIT55261.1 | GulD | BGC0000072 | Gulmirecin\_biosynthetic\_gene\_cluster | 33 | 43.4969032873 | 450 | 8.6e-126 |
| CAL58684.1 | polyketide\_synthase | BGC0000149 | Spirangien\_biosynthetic\_gene\_cluster | 33 | 44.1162458313 | 450 | 1.1e-125 |
| ACA99172.1 | Polyketide\_synthase | BGC0001160 | Nonadecene\_biosynthetic\_gene\_cluster | 33 | 43.6398284898 | 449 | 1.9e-125 |
| AGC45622.1 | polyketide\_synthase | BGC0001394 | Phenalamide\_biosynthetic\_gene\_cluster | 32 | 47.0700333492 | 448 | 3.3e-125 |
| WP\_042799407.1 | polyketide\_synthase | BGC0001283 | Arsenopolyketides\_biosynthetic\_gene\_cluster | 33 | 44.9737970462 | 448 | 3.3e-125 |
| CAD19091.1 | StiG\_protein | BGC0000153 | Stigmatellin\_biosynthetic\_gene\_cluster | 32 | 48.1181515007 | 448 | 4.3e-125 |
| AHA38201.1 | GphH | BGC0000069 | Gephyronic\_acid\_biosynthetic\_gene\_cluster | 34 | 43.5445450214 | 446 | 1.2e-124 |
| CCE88381.1 | polyketide\_synthase | BGC0001034 | Pellasoren\_biosynthetic\_gene\_cluster | 32 | 47.6893758933 | 446 | 1.6e-124 |
| AIT55263.1 | GulF | BGC0000072 | Gulmirecin\_biosynthetic\_gene\_cluster | 32 | 47.6417341591 | 446 | 2.1e-124 |
| CAD19088.1 | StiD\_protein | BGC0000153 | Stigmatellin\_biosynthetic\_gene\_cluster | 33 | 44.020962363 | 445 | 2.8e-124 |
| CCE88380.1 | polyketide\_synthase | BGC0001034 | Pellasoren\_biosynthetic\_gene\_cluster | 31 | 51.1672224869 | 445 | 2.8e-124 |
| AFU82615.1 | polyketide\_synthase | BGC0000998 | Guadinomine\_biosynthetic\_gene\_cluster | 32 | 43.1634111482 | 445 | 4.7e-124 |
| AAX98189.1 | polyketide\_synthase\_type\_I | BGC0000052 | ECO-02301\_biosynthetic\_gene\_cluster | 30 | 57.1224392568 | 444 | 6.2e-124 |
| AAS98781.1 | JamJ | BGC0001001 | Jamaicamide\_biosynthetic\_gene\_cluster | 29 | 49.2615531205 | 444 | 8e-124 |
| AAF26922.1 | polyketide\_synthase | BGC0000988 | Epothilone\_biosynthetic\_gene\_cluster | 31 | 50.7860886136 | 444 | 8e-124 |
| CAQ43076.1 | polyketide\_synthase | BGC0000970 | Chondrochloren\_biosynthetic\_gene\_cluster | 32 | 47.5464506908 | 443 | 1.4e-123 |
| CBD77734.1 | polyketide\_synthase | BGC0000974 | Crocacin\_biosynthetic\_gene\_cluster | 33 | 43.7827536922 | 443 | 1.4e-123 |
| CAD29793.1 | polyketide\_synthase\_type\_I | BGC0001015 | Microcystin\_biosynthetic\_gene\_cluster | 32 | 42.7822772749 | 442 | 2.3e-123 |
| CAO98850.1 | polyketide\_synthase\_AufG | BGC0000023 | Aurafuron\_biosynthetic\_gene\_cluster | 32 | 43.0204859457 | 442 | 2.3e-123 |
| AAF71766.1 | NysI | BGC0000115 | Nystatin\_biosynthetic\_gene\_cluster | 33 | 43.3539780848 | 442 | 2.3e-123 |
| ACB46196.1 | polyketide\_synthase | BGC0000989 | Epothilone\_biosynthetic\_gene\_cluster | 30 | 56.7889471177 | 441 | 5.2e-123 |
| AAQ82561.1 | FscA | BGC0000034 | Candicidin\_biosynthetic\_gene\_cluster | 33 | 43.6874702239 | 441 | 6.8e-123 |
| BAT51066.1 | type\_I\_polyketide\_synthase | BGC0001296 | Streptazone\_E\_biosynthetic\_gene\_cluster | 32 | 43.115769414 | 441 | 6.8e-123 |
| CAL58686.1 | polyketide\_synthase | BGC0000149 | Spirangien\_biosynthetic\_gene\_cluster | 31 | 50.9290138161 | 440 | 1.2e-122 |
| AAF62884.1 | EpoE | BGC0000991 | Epothilone\_biosynthetic\_gene\_cluster | 31 | 50.8813720819 | 439 | 2e-122 |
| CAO98879.1 | polyketide\_synthase\_AufD | BGC0000023 | Aurafuron\_biosynthetic\_gene\_cluster | 32 | 43.3063363506 | 438 | 3.4e-122 |
| ADB12492.1 | EpoE | BGC0000990 | Epothilone\_biosynthetic\_gene\_cluster | 31 | 50.8813720819 | 438 | 3.4e-122 |
| CCE88378.1 | polyketide\_synthase | BGC0001034 | Pellasoren\_biosynthetic\_gene\_cluster | 32 | 47.4035254883 | 438 | 3.4e-122 |
| AMB48441.1 | polyketide\_synthase | BGC0001357 | Carbamidocyclophane\_biosynthetic\_gene\_cluster | 30 | 43.1634111482 | 438 | 3.4e-122 |
| ADX66461.1 | ScnS2 | BGC0000108 | Natamycin\_biosynthetic\_gene\_cluster | 32 | 43.5445450214 | 438 | 4.4e-122 |
| CQR60493.1 | Polyketide\_synthase,\_type\_I,\_modules:\_9\_and\_10 | BGC0001287 | Chaxamycin\_A\_/\_chaxamycin\_B\_/\_chaxamycin\_C\_/\_chaxamycin\_D | 33 | 43.1634111482 | 437 | 7.5e-122 |
| AAK57188.1 | MxaD | BGC0001022 | Myxalamid\_biosynthetic\_gene\_cluster | 31 | 43.2110528823 | 437 | 9.8e-122 |
| AFV96142.1 | polyketide\_synthase | BGC0001064 | Cylindrocyclophane\_biosynthetic\_gene\_cluster | 31 | 43.1634111482 | 437 | 9.8e-122 |
| CAQ43078.1 | polyketide\_synthase | BGC0000970 | Chondrochloren\_biosynthetic\_gene\_cluster | 32 | 47.4988089566 | 437 | 9.8e-122 |
| AEP40939.1 | polyketide\_synthase\_type\_I | BGC0000021 | Apoptolidin\_biosynthetic\_gene\_cluster | 32 | 42.9728442115 | 436 | 1.3e-121 |
| ADH04639.1 | TgaA | BGC0001051 | Thuggacin\_biosynthetic\_gene\_cluster | 32 | 43.6874702239 | 436 | 1.3e-121 |
| ALD82522.1 | polyketide\_synthase | BGC0001212 | Nannocystin\_biosynthetic\_gene\_cluster | 32 | 44.1162458313 | 436 | 1.7e-121 |
| ADC79639.1 | TamAIII | BGC0001052 | Tirandamycin\_biosynthetic\_gene\_cluster | 32 | 43.8780371606 | 436 | 2.2e-121 |
| BAT51065.1 | type\_I\_polyketide\_synthase | BGC0001296 | Streptazone\_E\_biosynthetic\_gene\_cluster | 32 | 43.8780371606 | 435 | 2.9e-121 |
| CAA16183.1 | polyketide\_synthase | BGC0001063 | Undecylprodigiosin\_biosynthetic\_gene\_cluster | 33 | 42.9252024774 | 435 | 3.7e-121 |
| EED18001.1 | polyketide\_synthase,\_putative | BGC0000154 | Stipitatic\_acid\_biosynthetic\_gene\_cluster | 30 | 55.1214864221 | 434 | 8.3e-121 |
| AAF19810.1 | MtaB | BGC0001024 | Myxothiazol\_biosynthetic\_gene\_cluster | 31 | 49.9761791329 | 434 | 8.3e-121 |
| AAG23263.1 | polyketide\_synthase\_extender\_modules\_5-7 | BGC0000148 | Spinosad\_biosynthetic\_gene\_cluster | 33 | 43.401619819 | 433 | 1.1e-120 |
| CBD77732.1 | polyketide\_synthase | BGC0000974 | Crocacin\_biosynthetic\_gene\_cluster | 32 | 45.0214387804 | 433 | 1.9e-120 |
| AHN85651.1 | Phn2 | BGC0000122 | Phenylnannolone\_A\_biosynthetic\_gene\_cluster | 32 | 44.1638875655 | 432 | 2.4e-120 |
| CAC20921.1 | PimS2\_protein | BGC0000125 | Pimaricin\_biosynthetic\_gene\_cluster | 32 | 44.4973797046 | 432 | 2.4e-120 |
| ACY13415.1 | KR\_domain\_protein | BGC0001367 | Haliamide\_biosynthetic\_gene\_cluster | 32 | 43.2586946165 | 432 | 3.2e-120 |
| ADX66472.1 | ScnS1 | BGC0000108 | Natamycin\_biosynthetic\_gene\_cluster | 33 | 42.4487851358 | 431 | 5.4e-120 |
| EHA28237.1 | hypothetical\_protein | BGC0001143 | Azanigerone\_biosynthetic\_gene\_cluster | 25 | 75.1310147689 | 431 | 7e-120 |
| CBD77738.1 | polyketide\_synthase | BGC0000974 | Crocacin\_biosynthetic\_gene\_cluster | 33 | 43.115769414 | 430 | 9.2e-120 |
| AAQ90174.1 | polyketide\_synthase\_type\_I | BGC0000128 | Pyoluteorin\_biosynthetic\_gene\_cluster | 32 | 44.2115292997 | 430 | 1.6e-119 |
| AAF26919.1 | polyketide\_synthase | BGC0000988 | Epothilone\_biosynthetic\_gene\_cluster | 32 | 43.6398284898 | 429 | 2.7e-119 |
| AAS98784.1 | JamM | BGC0001001 | Jamaicamide\_biosynthetic\_gene\_cluster | 32 | 43.5445450214 | 428 | 3.5e-119 |
| AAT70102.1 | CurG | BGC0001165 | Curacin\_biosynthetic\_gene\_cluster | 32 | 43.6398284898 | 428 | 3.5e-119 |
| AEE88283.1 | CurG | BGC0000976 | Curacin\_biosynthetic\_gene\_cluster | 32 | 43.6398284898 | 428 | 3.5e-119 |
| ADH04658.1 | TugB | BGC0001342 | Thuggacin\_biosynthetic\_gene\_cluster | 32 | 43.3539780848 | 428 | 6e-119 |
| ACN69989.1 | polyketide\_synthase | BGC0000079 | Indanomycin\_biosynthetic\_gene\_cluster | 32 | 42.9252024774 | 427 | 7.8e-119 |
| CAQ18830.1 | polyketide\_synthase | BGC0000954 | Ajudazol\_biosynthetic\_gene\_cluster | 31 | 50.9766555503 | 427 | 1e-118 |
| AAK73501.1 | AmphI | BGC0000015 | Amphotericin\_biosynthetic\_gene\_cluster | 32 | 44.5926631729 | 427 | 1e-118 |
| AGC45624.1 | polyketide\_synthase | BGC0001394 | Phenalamide\_biosynthetic\_gene\_cluster | 32 | 43.3539780848 | 426 | 1.3e-118 |
| AAK57186.1 | MxaB2 | BGC0001022 | Myxalamid\_biosynthetic\_gene\_cluster | 33 | 41.305383516 | 426 | 1.3e-118 |
| CAC20931.1 | PimS1\_protein | BGC0000125 | Pimaricin\_biosynthetic\_gene\_cluster | 32 | 43.2586946165 | 426 | 1.3e-118 |
| CAQ43077.1 | polyketide\_synthase | BGC0000970 | Chondrochloren\_biosynthetic\_gene\_cluster | 31 | 48.8327775131 | 426 | 2.3e-118 |
| BAD38874.1 | polyketide\_synthase | BGC0000111 | Neocarzilin\_biosynthetic\_gene\_cluster | 33 | 42.7346355407 | 425 | 3.9e-118 |
| AEE88284.1 | CurF | BGC0000976 | Curacin\_biosynthetic\_gene\_cluster | 29 | 52.7870414483 | 425 | 3.9e-118 |
| AAF00958.1 | McyE | BGC0001017 | Microcystin\_biosynthetic\_gene\_cluster | 32 | 43.2110528823 | 425 | 5e-118 |
| AKD43522.1 | Type\_I\_polyketide\_synthase | BGC0001409 | Dutomycin\_biosynthetic\_gene\_cluster | 31 | 47.594092425 | 424 | 6.6e-118 |
| AAF62880.1 | EpoA | BGC0000991 | Epothilone\_biosynthetic\_gene\_cluster | 32 | 43.6398284898 | 424 | 6.6e-118 |
| BAQ25511.1 | type\_I\_polyketide\_synthase | BGC0001288 | Maklamicin\_biosynthetic\_gene\_cluster | 31 | 43.7351119581 | 424 | 6.6e-118 |
| BAJ16467.1 | polyketide\_synthase | BGC0000058 | FD-891\_biosynthetic\_gene\_cluster | 29 | 56.9795140543 | 424 | 8.6e-118 |
| CBA11582.1 | polyketide\_synthase\_type\_I | BGC0001046 | Streptolydigin\_biosynthetic\_gene\_cluster | 30 | 48.3087184374 | 424 | 8.6e-118 |
| CAC22145.1 | putative\_type\_I\_polyketide\_synthase | BGC0000038 | Coelimycin\_biosynthetic\_gene\_cluster | 31 | 42.9728442115 | 424 | 8.6e-118 |
| ADB12488.1 | EpoA | BGC0000990 | Epothilone\_biosynthetic\_gene\_cluster | 32 | 43.6398284898 | 423 | 1.1e-117 |
| ACB46192.1 | polyketide\_synthase | BGC0000989 | Epothilone\_biosynthetic\_gene\_cluster | 32 | 43.6398284898 | 423 | 1.5e-117 |
| ADH04660.1 | TugD | BGC0001342 | Thuggacin\_biosynthetic\_gene\_cluster | 32 | 43.6398284898 | 423 | 1.5e-117 |
| AKD43768.1 | HerA1 | BGC0001349 | Heronamide\_biosynthetic\_gene\_cluster | 29 | 47.2606002859 | 423 | 1.5e-117 |
| AAT70101.1 | CurF | BGC0001165 | Curacin\_biosynthetic\_gene\_cluster | 29 | 52.7870414483 | 423 | 1.5e-117 |
| AAQ82568.1 | FscD | BGC0000061 | FR-008\_biosynthetic\_gene\_cluster | 31 | 44.306812768 | 423 | 1.9e-117 |
| AAF19812.1 | MtaD | BGC0001024 | Myxothiazol\_biosynthetic\_gene\_cluster | 29 | 48.3563601715 | 422 | 2.5e-117 |
| CAQ52624.1 | type\_I\_polyketide\_synthase,\_modules\_7-8 | BGC0001066 | Kendomycin\_biosynthetic\_gene\_cluster | 31 | 44.7355883754 | 422 | 2.5e-117 |
| AEH42490.1 | polyketide\_synthase | BGC0000032 | Calcimycin\_biosynthetic\_gene\_cluster | 32 | 42.6869938066 | 422 | 3.3e-117 |
| AHF22854.1 | MarL | BGC0000091 | Marineosin\_biosynthetic\_gene\_cluster | 33 | 43.2586946165 | 422 | 3.3e-117 |
| CAD29794.1 | peptide\_synthetase | BGC0001015 | Microcystin\_biosynthetic\_gene\_cluster | 32 | 42.6393520724 | 421 | 4.3e-117 |
| AAW03327.1 | CtaD | BGC0000982 | Cystothiazole\_A\_biosynthetic\_gene\_cluster | 27 | 60.6479275846 | 421 | 4.3e-117 |
| AFL48529.1 | laidlomycin\_polyketide\_synthase\_(module\_5\_and\_module\_6) | BGC0000084 | Laidlomycin\_biosynthetic\_gene\_cluster | 32 | 42.4011434016 | 421 | 5.6e-117 |
| AEP40940.1 | polyketide\_synthase\_type\_I | BGC0000021 | Apoptolidin\_biosynthetic\_gene\_cluster | 33 | 43.3539780848 | 421 | 5.6e-117 |
| AFI57005.1 | QmnA1 | BGC0000133 | Quartromicin\_biosynthetic\_gene\_cluster | 33 | 42.4011434016 | 421 | 5.6e-117 |
| AEP40935.1 | polyketide\_synthase\_type\_I | BGC0000021 | Apoptolidin\_biosynthetic\_gene\_cluster | 32 | 42.1629347308 | 421 | 7.3e-117 |
| BAO66542.1 | type\_I\_polyketide\_synthase | BGC0000042 | Cremimycin\_biosynthetic\_gene\_cluster | 33 | 43.5921867556 | 421 | 7.3e-117 |
| AAW03325.1 | CtaB | BGC0000982 | Cystothiazole\_A\_biosynthetic\_gene\_cluster | 31 | 44.9261553121 | 420 | 9.5e-117 |
| ACR50791.1 | putative\_polyketide\_synthase | BGC0000163 | Tetronasin\_biosynthetic\_gene\_cluster | 31 | 42.5917103383 | 420 | 9.5e-117 |
| ABO15861.1 | polyketide\_synthase | BGC0000130 | Pyrrolomycin\_biosynthetic\_gene\_cluster | 33 | 43.0204859457 | 420 | 9.5e-117 |
| CAJ46689.1 | polyketide\_synthase | BGC0000969 | Chondramide\_biosynthetic\_gene\_cluster | 31 | 51.4054311577 | 420 | 1.2e-116 |
| BAQ25512.1 | type\_I\_polyketide\_synthase | BGC0001288 | Maklamicin\_biosynthetic\_gene\_cluster | 29 | 47.6417341591 | 419 | 2.8e-116 |
| BAG84248.1 | putative\_polyketide\_synthase | BGC0000257 | Prodigiosin\_biosynthetic\_gene\_cluster | 31 | 44.0686040972 | 419 | 2.8e-116 |
| CQR60495.1 | Polyketide\_synthase,\_type\_I,\_module\_7 | BGC0001287 | Chaxamycin\_A\_/\_chaxamycin\_B\_/\_chaxamycin\_C\_/\_chaxamycin\_D | 33 | 42.4964268699 | 418 | 3.6e-116 |
| CAJ46690.1 | polyketide\_synthase | BGC0000969 | Chondramide\_biosynthetic\_gene\_cluster | 31 | 48.7374940448 | 418 | 6.2e-116 |
| ADH04640.1 | TgaB | BGC0001051 | Thuggacin\_biosynthetic\_gene\_cluster | 31 | 44.4497379705 | 417 | 8.1e-116 |
| AEZ64504.1 | Herc | BGC0001065 | Herboxidiene\_biosynthetic\_gene\_cluster | 31 | 44.3544545021 | 417 | 8.1e-116 |
| AAF71776.1 | NysC | BGC0000115 | Nystatin\_biosynthetic\_gene\_cluster | 32 | 42.5440686041 | 417 | 1.1e-115 |
| BAH02268.1 | polyketide\_synthase | BGC0000126 | Pladienolide\_biosynthetic\_gene\_cluster | 30 | 48.0705097666 | 416 | 1.8e-115 |
| BAH02269.1 | polyketide\_synthase | BGC0000126 | Pladienolide\_biosynthetic\_gene\_cluster | 31 | 43.9256788947 | 416 | 1.8e-115 |
| AAZ77698.1 | ChlA5 | BGC0000036 | Chlorothricin\_biosynthetic\_gene\_cluster | 31 | 46.9747498809 | 416 | 1.8e-115 |
| ANI24099.1 | polyketide\_synthase | BGC0001235 | Antalid\_biosynthetic\_gene\_cluster | 32 | 49.3091948547 | 416 | 1.8e-115 |
| AEH42491.1 | polyketide\_synthase | BGC0000032 | Calcimycin\_biosynthetic\_gene\_cluster | 32 | 42.8775607432 | 416 | 2.3e-115 |
| BAO66539.1 | type\_I\_polyketide\_synthase | BGC0000042 | Cremimycin\_biosynthetic\_gene\_cluster | 32 | 43.9256788947 | 416 | 2.3e-115 |
| BAH02271.1 | polyketide\_synthase | BGC0000126 | Pladienolide\_biosynthetic\_gene\_cluster | 31 | 43.6874702239 | 416 | 2.3e-115 |
| ADB12493.1 | EpoF | BGC0000990 | Epothilone\_biosynthetic\_gene\_cluster | 33 | 43.5445450214 | 416 | 2.3e-115 |
| AAF62885.1 | EpoF | BGC0000991 | Epothilone\_biosynthetic\_gene\_cluster | 33 | 43.7351119581 | 415 | 3.1e-115 |
| BAQ25483.1 | type\_I\_polyketide\_synthase | BGC0001288 | Maklamicin\_biosynthetic\_gene\_cluster | 31 | 44.0686040972 | 415 | 3.1e-115 |
| AAF26923.1 | polyketide\_synthase | BGC0000988 | Epothilone\_biosynthetic\_gene\_cluster | 33 | 43.7351119581 | 415 | 4e-115 |
| AEZ53946.1 | polyketide\_synthase | BGC0000144 | Salinomycin\_biosynthetic\_gene\_cluster | 31 | 47.8799428299 | 415 | 4e-115 |
| ABB05104.1 | LipPks3 | BGC0001003 | Lipomycin\_biosynthetic\_gene\_cluster | 29 | 48.8804192473 | 415 | 4e-115 |
| AIT55259.1 | GulB | BGC0000072 | Gulmirecin\_biosynthetic\_gene\_cluster | 33 | 43.4492615531 | 415 | 4e-115 |
| WP\_063764077.1 | hypothetical\_protein | BGC0001348 | JBIR-100\_biosynthetic\_gene\_cluster | 30 | 47.6893758933 | 415 | 4e-115 |
| AEC13080.1 | fosB | BGC0000060 | Fostriecin\_biosynthetic\_gene\_cluster | 31 | 44.020962363 | 414 | 6.8e-115 |
| CAC22144.1 | putative\_type\_I\_polyketide\_synthase | BGC0000038 | Coelimycin\_biosynthetic\_gene\_cluster | 31 | 42.6869938066 | 414 | 6.8e-115 |
| BAJ16470.1 | polyketide\_synthase | BGC0000058 | FD-891\_biosynthetic\_gene\_cluster | 29 | 58.8375416865 | 414 | 6.8e-115 |
| AAZ77697.1 | ChlA4 | BGC0000036 | Chlorothricin\_biosynthetic\_gene\_cluster | 30 | 47.3558837542 | 414 | 8.9e-115 |
| CAD89773.1 | MelB\_protein | BGC0001009 | Melithiazol\_biosynthetic\_gene\_cluster | 31 | 45.5454978561 | 414 | 8.9e-115 |
| BAJ16468.1 | polyketide\_synthase | BGC0000058 | FD-891\_biosynthetic\_gene\_cluster | 31 | 43.401619819 | 414 | 8.9e-115 |
| AHA38203.1 | GphJ | BGC0000069 | Gephyronic\_acid\_biosynthetic\_gene\_cluster | 32 | 44.1162458313 | 413 | 1.2e-114 |
| AHH99921.1 | PKS\_I | BGC0000002 | Aculeximycin\_biosynthetic\_gene\_cluster | 33 | 42.210576465 | 413 | 1.2e-114 |
| BAC68129.1 | modular\_polyketide\_synthase | BGC0000059 | Filipin\_biosynthetic\_gene\_cluster | 30 | 47.5464506908 | 413 | 1.2e-114 |
| AHH99920.1 | PKS\_I | BGC0000002 | Aculeximycin\_biosynthetic\_gene\_cluster | 32 | 42.4011434016 | 413 | 1.2e-114 |
| ACB46197.1 | polyketide\_synthase | BGC0000989 | Epothilone\_biosynthetic\_gene\_cluster | 33 | 43.7351119581 | 413 | 1.2e-114 |
| AAZ77693.1 | ChlA1 | BGC0000036 | Chlorothricin\_biosynthetic\_gene\_cluster | 31 | 48.1657932349 | 413 | 2e-114 |
| BAF02924.1 | type\_I\_polyketide\_synthase | BGC0000073 | Halstoctacosanolide\_biosynthetic\_gene\_cluster | 31 | 43.2110528823 | 413 | 2e-114 |
| ABK32256.1 | AmbB | BGC0000014 | Ambruticin\_biosynthetic\_gene\_cluster | 32 | 43.4492615531 | 413 | 2e-114 |
| AAK73514.1 | AmphC | BGC0000015 | Amphotericin\_biosynthetic\_gene\_cluster | 29 | 46.8318246784 | 412 | 2.6e-114 |
| AEZ53947.1 | polyketide\_synthase | BGC0000144 | Salinomycin\_biosynthetic\_gene\_cluster | 32 | 42.7822772749 | 412 | 2.6e-114 |
| AHH99919.1 | PKS\_I | BGC0000002 | Aculeximycin\_biosynthetic\_gene\_cluster | 32 | 42.3058599333 | 412 | 3.4e-114 |
| BAE93729.1 | type\_I\_polyketide\_synthase | BGC0000164 | Tetronomycin\_biosynthetic\_gene\_cluster | 31 | 43.5445450214 | 412 | 3.4e-114 |
| AEZ54378.1 | PieA5 | BGC0000124 | Piericidin\_A1\_biosynthetic\_gene\_cluster | 30 | 47.9752262982 | 412 | 3.4e-114 |
| ABK32288.1 | JerB | BGC0000080 | Jerangolid\_biosynthetic\_gene\_cluster | 32 | 42.7346355407 | 412 | 3.4e-114 |
| WP\_043476516.1 | beta-ketoacyl\_synthase | BGC0001232 | Leucanicidin\_biosynthetic\_gene\_cluster | 30 | 48.7851357789 | 411 | 4.4e-114 |
| CAM00062.1 | EryAI\_Erythromycin\_polyketide\_synthase\_modules\_1\_and\_2 | BGC0000055 | Erythromycin\_biosynthetic\_gene\_cluster | 33 | 42.8299190091 | 411 | 4.4e-114 |
| BAF02926.1 | type\_I\_polyketide\_synthase | BGC0000073 | Halstoctacosanolide\_biosynthetic\_gene\_cluster | 32 | 44.2591710338 | 411 | 5.8e-114 |
| ABC84471.1 | NigAVII | BGC0000114 | Nigericin\_biosynthetic\_gene\_cluster | 31 | 45.0214387804 | 411 | 5.8e-114 |
| CCP20051.1 | divM\_protein | BGC0001119 | Divergolide\_biosynthetic\_gene\_cluster | 31 | 47.0700333492 | 411 | 5.8e-114 |
| CUW01176.1 | Type\_I\_polyketide\_synthase | BGC0001359\_c2 | PM100117\_/\_PM100118\_biosynthetic\_gene\_cluster | 31 | 44.1638875655 | 411 | 5.8e-114 |
| ACR50773.1 | polyketide\_synthase | BGC0000163 | Tetronasin\_biosynthetic\_gene\_cluster | 30 | 47.2606002859 | 411 | 7.5e-114 |
| AAG23265.1 | polyketide\_synthase\_extender\_module\_2 | BGC0000148 | Spinosad\_biosynthetic\_gene\_cluster | 31 | 43.7827536922 | 410 | 9.8e-114 |
| AGY30676.1 | Ann4 | BGC0001298 | 4-Z-annimycin\_biosynthetic\_gene\_cluster | 32 | 42.3058599333 | 410 | 9.8e-114 |
| ABW96540.1 | type\_I\_modular\_polyketide\_synthase | BGC0000159 | Tautomycin\_biosynthetic\_gene\_cluster | 32 | 43.3539780848 | 410 | 9.8e-114 |
| BAO66519.1 | type\_I\_polyketide\_synthase | BGC0000042 | Cremimycin\_biosynthetic\_gene\_cluster | 32 | 42.4487851358 | 410 | 9.8e-114 |
| ABL86391.1 | hybrid\_polyketide\_synthase\_and\_nonribosomal\_peptide\_synthetase | BGC0000999 | Heat-stable\_antifungal\_factor\_biosynthetic\_gene\_cluster | 32 | 43.401619819 | 410 | 1.3e-113 |
| ABV83221.1 | CppI | BGC0000116 | Nystatin-like\_Pseudonocardia\_polyene\_biosynthetic\_gene\_cluster | 31 | 43.115769414 | 410 | 1.3e-113 |
| AEP40936.1 | polyketide\_synthase\_type\_I | BGC0000021 | Apoptolidin\_biosynthetic\_gene\_cluster | 32 | 42.7346355407 | 410 | 1.7e-113 |
| BAO66541.1 | type\_I\_polyketide\_synthase | BGC0000042 | Cremimycin\_biosynthetic\_gene\_cluster | 32 | 42.7346355407 | 410 | 1.7e-113 |
| AEZ54379.1 | PieA6 | BGC0000124 | Piericidin\_A1\_biosynthetic\_gene\_cluster | 31 | 42.8775607432 | 410 | 1.7e-113 |
| AHB82059.1 | non\_ribosomal\_peptide\_synthetase/polyketide\_synthase | BGC0001019 | Microsclerodermins\_biosynthetic\_gene\_cluster | 32 | 43.8303954264 | 409 | 2.2e-113 |
| AFL48528.1 | laidlomycin\_polyketide\_synthase\_(module\_7\_and\_module\_8) | BGC0000084 | Laidlomycin\_biosynthetic\_gene\_cluster | 32 | 42.6869938066 | 409 | 2.9e-113 |
| ALA62323.1 | methylphloroacetophenone\_synthase | BGC0001309 | Usnic\_acid\_biosynthetic\_gene\_cluster | 30 | 56.884230586 | 409 | 2.9e-113 |
| ABK32259.1 | AmbE | BGC0000014 | Ambruticin\_biosynthetic\_gene\_cluster | 32 | 43.0204859457 | 408 | 3.7e-113 |
| CAD17792.1 | probable\_non\_ribosomal\_peptide\_synthetase\_protein | BGC0001363 | Ralsolamycin\_biosynthetic\_gene\_cluster | 32 | 43.9256788947 | 408 | 3.7e-113 |
| CAQ43075.1 | polyketide\_synthase | BGC0000970 | Chondrochloren\_biosynthetic\_gene\_cluster | 30 | 52.3582658409 | 408 | 3.7e-113 |
| AEU17899.1 | putative\_type\_I\_PKS | BGC0001072 | Simocyclinone\_biosynthetic\_gene\_cluster | 31 | 44.3544545021 | 408 | 3.7e-113 |
| BAF02921.1 | type\_I\_polyketide\_synthase | BGC0000073 | Halstoctacosanolide\_biosynthetic\_gene\_cluster | 30 | 44.9737970462 | 408 | 4.9e-113 |
| BAF02922.1 | type\_I\_polyketide\_synthase | BGC0000073 | Halstoctacosanolide\_biosynthetic\_gene\_cluster | 32 | 42.9728442115 | 408 | 4.9e-113 |
| BAC68126.1 | modular\_polyketide\_synthase | BGC0000059 | Filipin\_biosynthetic\_gene\_cluster | 32 | 42.6393520724 | 408 | 4.9e-113 |
| ABB05105.1 | LipPks4 | BGC0001003 | Lipomycin\_biosynthetic\_gene\_cluster | 31 | 43.9256788947 | 408 | 6.4e-113 |
| CAL58683.1 | polyketide\_synthase | BGC0000149 | Spirangien\_biosynthetic\_gene\_cluster | 31 | 43.9733206289 | 407 | 8.3e-113 |
| AAX98186.1 | polyketide\_synthase\_type\_I | BGC0000052 | ECO-02301\_biosynthetic\_gene\_cluster | 32 | 42.8775607432 | 406 | 2.4e-112 |
| AAN32979.1 | BarE | BGC0000962 | Barbamide\_biosynthetic\_gene\_cluster | 31 | 44.2591710338 | 406 | 2.4e-112 |
| BAF85844.1 | modular\_polyketide\_synthase | BGC0000109 | Nemadectin\_biosynthetic\_gene\_cluster | 31 | 43.6874702239 | 405 | 3.2e-112 |
| BAR73017.1 | putative\_PKS\_(KS-AT-KR-ACP-KS-AT-DH-KR-ACP) | BGC0001194 | Hitachimycin\_biosynthetic\_gene\_cluster | 32 | 42.8299190091 | 405 | 3.2e-112 |
| BAQ25513.1 | type\_I\_polyketide\_synthase | BGC0001288 | Maklamicin\_biosynthetic\_gene\_cluster | 30 | 48.0705097666 | 405 | 4.1e-112 |
| AAY28227.1 | HbmAIII | BGC0000074 | Herbimycin\_biosynthetic\_gene\_cluster | 32 | 44.2115292997 | 405 | 4.1e-112 |
| ABB05103.1 | LipPks2 | BGC0001003 | Lipomycin\_biosynthetic\_gene\_cluster | 30 | 47.0223916151 | 405 | 4.1e-112 |
| ALA09355.1 | type\_I\_modular\_PKS | BGC0001303 | Lobosamide\_biosynthetic\_gene\_cluster | 32 | 43.0204859457 | 404 | 5.4e-112 |
| AAS98787.1 | JamP | BGC0001001 | Jamaicamide\_biosynthetic\_gene\_cluster | 32 | 42.9728442115 | 404 | 5.4e-112 |
| AAZ77696.1 | ChlA3 | BGC0000036 | Chlorothricin\_biosynthetic\_gene\_cluster | 32 | 42.8299190091 | 404 | 5.4e-112 |
| ACY06288.1 | type\_I\_polyketide\_synthase | BGC0001042 | Sanglifehrin\_A\_biosynthetic\_gene\_cluster | 31 | 47.2129585517 | 404 | 7.1e-112 |
| BAF02923.1 | type\_I\_polyketide\_synthase | BGC0000073 | Halstoctacosanolide\_biosynthetic\_gene\_cluster | 32 | 42.8299190091 | 404 | 9.2e-112 |
| CAQ43079.1 | polyketide\_synthase | BGC0000970 | Chondrochloren\_biosynthetic\_gene\_cluster | 32 | 44.8785135779 | 403 | 1.2e-111 |
| ACB46486.1 | polyketide\_synthase | BGC0000082 | Kijanimicin\_biosynthetic\_gene\_cluster | 31 | 47.5464506908 | 403 | 1.2e-111 |
| BAJ16469.1 | polyketide\_synthase | BGC0000058 | FD-891\_biosynthetic\_gene\_cluster | 31 | 44.2115292997 | 403 | 1.2e-111 |
| BAD08373.1 | polyketide\_synthase\_modules\_1-3 | BGC0000167 | Vicenistatin\_biosynthetic\_gene\_cluster | 32 | 43.1634111482 | 403 | 1.6e-111 |
| ABJ97437.1 | MerA | BGC0001012 | Meridamycin\_biosynthetic\_gene\_cluster | 30 | 48.213434969 | 403 | 2.1e-111 |
| AAA79984.2 | soraphen\_polyketide\_synthase\_B | BGC0000147 | Soraphen\_biosynthetic\_gene\_cluster | 31 | 43.6398284898 | 403 | 2.1e-111 |
| ABK32257.1 | AmbC | BGC0000014 | Ambruticin\_biosynthetic\_gene\_cluster | 31 | 48.1657932349 | 403 | 2.1e-111 |
| BAH02270.1 | polyketide\_synthase | BGC0000126 | Pladienolide\_biosynthetic\_gene\_cluster | 31 | 43.5445450214 | 403 | 2.1e-111 |
| CUW01175.1 | Type\_I\_polyketide\_synthase | BGC0001359 | PM100117\_/\_PM100118\_biosynthetic\_gene\_cluster | 31 | 43.9256788947 | 402 | 2.7e-111 |
| AAO06918.1 | GdmAIII | BGC0000066 | Geldanamycin\_biosynthetic\_gene\_cluster | 31 | 44.1162458313 | 402 | 3.5e-111 |
| AAP42873.1 | NanA11 | BGC0000105 | Nanchangmycin\_biosynthetic\_gene\_cluster | 31 | 49.7856121963 | 402 | 3.5e-111 |
| ABB86410.1 | GelC | BGC0000067 | Geldanamycin\_biosynthetic\_gene\_cluster | 31 | 44.1162458313 | 402 | 3.5e-111 |
| CCP20047.1 | divK\_protein | BGC0001119 | Divergolide\_biosynthetic\_gene\_cluster | 31 | 46.3554073368 | 401 | 6e-111 |
| AFL48527.1 | laidlomycin\_polyketide\_synthase\_(module\_3\_and\_module\_4) | BGC0000084 | Laidlomycin\_biosynthetic\_gene\_cluster | 31 | 45.2596474512 | 401 | 6e-111 |
| AFU82614.1 | mixed\_NRPS\_PKS | BGC0000998 | Guadinomine\_biosynthetic\_gene\_cluster | 30 | 44.7355883754 | 401 | 7.8e-111 |
| AAF71774.1 | NysA | BGC0000115 | Nystatin\_biosynthetic\_gene\_cluster | 32 | 42.5440686041 | 401 | 7.8e-111 |
| CAJ88176.1 | putative\_polyketide\_synthase\_B | BGC0000151 | Stambomycin\_biosynthetic\_gene\_cluster | 31 | 43.9256788947 | 400 | 1e-110 |
| ABK32289.1 | JerC | BGC0000080 | Jerangolid\_biosynthetic\_gene\_cluster | 31 | 48.1657932349 | 400 | 1e-110 |
| AAO65799.1 | monensin\_polyketide\_synthase\_modules\_5\_and\_6 | BGC0000100 | Monensin\_biosynthetic\_gene\_cluster | 30 | 47.0223916151 | 400 | 1e-110 |
| CUW01173.1 | Type\_I\_polyketide\_synthase | BGC0001359 | PM100117\_/\_PM100118\_biosynthetic\_gene\_cluster | 31 | 50.5478799428 | 400 | 1.3e-110 |
| AAC69329.1 | type\_I\_polyketide\_synthase\_PikAI | BGC0000094 | Methymycin\_/\_pikromycin\_biosynthetic\_gene\_cluster | 32 | 43.7351119581 | 400 | 1.3e-110 |
| AAP42858.1 | NanA4 | BGC0000105 | Nanchangmycin\_biosynthetic\_gene\_cluster | 31 | 43.9733206289 | 400 | 1.3e-110 |
| ABI91466.1 | beta-ketoacyl\_synthase | BGC0001094 | Enacyloxin\_biosynthetic\_gene\_cluster | 31 | 42.6869938066 | 400 | 1.3e-110 |
| AHA38199.1 | GphF | BGC0000069 | Gephyronic\_acid\_biosynthetic\_gene\_cluster | 32 | 44.0686040972 | 399 | 1.7e-110 |
| BAQ21947.1 | putative\_type\_I\_polyketide\_synthase | BGC0001204 | Versipelostatin\_biosynthetic\_gene\_cluster | 30 | 48.7851357789 | 399 | 1.7e-110 |
| ALP32043.1 | CycC | BGC0001293 | Cyclizidine\_biosynthetic\_gene\_cluster | 31 | 47.3558837542 | 399 | 1.7e-110 |
| AJO72737.1 | Type\_I\_modular\_polyketide\_synthase | BGC0001381 | Brasilinolide\_biosynthetic\_gene\_cluster | 31 | 43.7827536922 | 399 | 1.7e-110 |
| BAF02927.1 | type\_I\_polyketide\_synthase | BGC0000073 | Halstoctacosanolide\_biosynthetic\_gene\_cluster | 31 | 43.401619819 | 399 | 1.7e-110 |
| AFP87524.1 | type\_I\_polyketide\_synthase | BGC0001159 | Marinopyrrole\_biosynthetic\_gene\_cluster | 31 | 43.4492615531 | 399 | 1.7e-110 |
| AGI61662.1 | modular\_PKS | BGC0000095\_c5 | Micromonolactam\_biosynthetic\_gene\_cluster | 31 | 43.3063363506 | 399 | 1.7e-110 |
| BAF02925.1 | type\_I\_polyketide\_synthase | BGC0000073 | Halstoctacosanolide\_biosynthetic\_gene\_cluster | 31 | 43.3063363506 | 399 | 1.7e-110 |
| ADU86004.1 | putative\_modular\_polyketide\_synthase | BGC0000165 | Tiacumicin\_B\_biosynthetic\_gene\_cluster | 32 | 42.3535016675 | 399 | 2.3e-110 |
| ADH01663.1 | putative\_polyketide\_synthase\_PKS3 | BGC0000099 | Monascorubrin\_biosynthetic\_gene\_cluster | 27 | 59.6474511672 | 399 | 2.3e-110 |
| AHH99925.1 | PKS\_I | BGC0000002 | Aculeximycin\_biosynthetic\_gene\_cluster | 32 | 42.3535016675 | 399 | 3e-110 |
| BAC76493.1 | lankamycin\_synthase\_LkmAI | BGC0000085 | Lankamycin\_biosynthetic\_gene\_cluster | 31 | 44.9261553121 | 399 | 3e-110 |
| AEK75502.1 | type\_1\_polyketide\_synthase | BGC0000001 | Abyssomicin\_biosynthetic\_gene\_cluster | 31 | 43.7351119581 | 398 | 3.9e-110 |
| BAR73021.1 | putative\_PKS\_(KS-AT-KR-ACP) | BGC0001194 | Hitachimycin\_biosynthetic\_gene\_cluster | 31 | 42.6869938066 | 398 | 3.9e-110 |
| AIT55262.1 | GulE | BGC0000072 | Gulmirecin\_biosynthetic\_gene\_cluster | 32 | 44.6879466413 | 398 | 3.9e-110 |
| ABB88534.1 | polyketide\_synthase\_type\_I | BGC0000049 | E-492\_/\_E-975\_biosynthetic\_gene\_cluster | 32 | 43.3063363506 | 398 | 6.6e-110 |
| AAM81585.1 | putative\_type\_I\_polyketide\_synthase | BGC0000047 | Dihydrochalcomycin\_biosynthetic\_gene\_cluster | 28 | 58.980466889 | 397 | 8.6e-110 |
| AAG13918.1 | megalomicin\_6-deoxyerythronolide\_B\_synthase\_2 | BGC0000092 | Megalomicin\_biosynthetic\_gene\_cluster | 31 | 45.1643639828 | 397 | 8.6e-110 |
| BAE93722.1 | type\_I\_polyketide\_synthase | BGC0000164 | Tetronomycin\_biosynthetic\_gene\_cluster | 32 | 44.8785135779 | 397 | 1.1e-109 |
| BAQ21939.1 | putative\_type\_I\_polyketide\_synthase | BGC0001204 | Versipelostatin\_biosynthetic\_gene\_cluster | 30 | 47.7370176274 | 397 | 1.1e-109 |
| AEU11005.1 | NpnA | BGC0001029 | Nostophycin\_biosynthetic\_gene\_cluster | 30 | 44.1162458313 | 397 | 1.1e-109 |
| ctg1\_orf521 |  | BGC0001199 | Akaeolide\_biosynthetic\_gene\_cluster | 32 | 43.0681276798 | 396 | 1.5e-109 |
| ADC45534.1 | modular\_polyketide\_synthase | BGC0000093 | Meilingmycin\_biosynthetic\_gene\_cluster | 29 | 44.4973797046 | 396 | 1.9e-109 |
| BAG17643.1 | putative\_NRPS-type-I\_PKS\_fusion\_protein | BGC0001043 | SGR\_PTMs\_biosynthetic\_gene\_cluster | 31 | 43.3539780848 | 396 | 1.9e-109 |
| CAQ18839.1 | hybrid\_polyketide\_synthase/nonribosomal\_polypetide\_synthetase | BGC0000954 | Ajudazol\_biosynthetic\_gene\_cluster | 32 | 43.7351119581 | 396 | 1.9e-109 |
| AAG23266.1 | polyketide\_synthase\_extender\_modules\_3-4 | BGC0000148 | Spinosad\_biosynthetic\_gene\_cluster | 31 | 42.6869938066 | 396 | 1.9e-109 |
| ADB12490.1 | EpoC | BGC0000990 | Epothilone\_biosynthetic\_gene\_cluster | 29 | 47.7370176274 | 396 | 2.5e-109 |
| CAE46851.1 | Type\_I\_modular\_polyketide\_synthase | BGC0000103 | Mycolactone\_biosynthetic\_gene\_cluster | 30 | 53.0252501191 | 396 | 2.5e-109 |
| CAE46843.1 | Type\_I\_modular\_polyketide\_synthase | BGC0000103 | Mycolactone\_biosynthetic\_gene\_cluster | 30 | 53.0252501191 | 396 | 2.5e-109 |
| ABC87512.1 | polyketide\_synthase | BGC0001011 | Meridamycin\_biosynthetic\_gene\_cluster | 31 | 43.3063363506 | 395 | 4.3e-109 |
| ctg1\_orf23 |  | BGC0001013 | Meridamycin\_biosynthetic\_gene\_cluster | 31 | 43.3063363506 | 395 | 4.3e-109 |
| ACB46487.1 | polyketide\_synthase | BGC0000082 | Kijanimicin\_biosynthetic\_gene\_cluster | 31 | 42.3535016675 | 395 | 4.3e-109 |
| AAZ77673.1 | ChlB1 | BGC0000036 | Chlorothricin\_biosynthetic\_gene\_cluster | 33 | 43.115769414 | 394 | 5.6e-109 |
| AAF26920.1 | polyketide\_synthase | BGC0000988 | Epothilone\_biosynthetic\_gene\_cluster | 29 | 47.7370176274 | 394 | 5.6e-109 |
| AAC01710.1 | RifA | BGC0000136 | Rifamycin\_biosynthetic\_gene\_cluster | 31 | 42.4487851358 | 394 | 7.3e-109 |
| AAF62882.1 | EpoC | BGC0000991 | Epothilone\_biosynthetic\_gene\_cluster | 29 | 47.7370176274 | 394 | 7.3e-109 |
| BAQ25481.1 | type\_I\_polyketide\_synthase | BGC0001288 | Maklamicin\_biosynthetic\_gene\_cluster | 30 | 44.2115292997 | 394 | 7.3e-109 |
| AFI57006.1 | QmnA2 | BGC0000133 | Quartromicin\_biosynthetic\_gene\_cluster | 31 | 47.9752262982 | 394 | 9.5e-109 |
| ANC94965.1 | AlmHII | BGC0001396 | Aldgamycin\_biosynthetic\_gene\_cluster | 27 | 58.8851834207 | 394 | 9.5e-109 |
| AAO06917.1 | GdmAII | BGC0000066 | Geldanamycin\_biosynthetic\_gene\_cluster | 28 | 57.9799904717 | 394 | 9.5e-109 |
| AHB82051.1 | polyketide\_synthase | BGC0001019 | Microsclerodermins\_biosynthetic\_gene\_cluster | 32 | 43.6398284898 | 394 | 9.5e-109 |
| AAF15892.2 | NosB | BGC0001028 | Nostopeptolide\_biosynthetic\_gene\_cluster | 31 | 43.8303954264 | 394 | 9.5e-109 |
| CAD89775.1 | MelD\_protein | BGC0001010 | Melithiazol\_biosynthetic\_gene\_cluster | 28 | 47.8323010958 | 393 | 1.2e-108 |
| CAE45669.1 | borrelidin\_polyketide\_synthase,\_type\_I | BGC0000031 | Borrelidin\_biosynthetic\_gene\_cluster | 31 | 43.0681276798 | 393 | 1.2e-108 |
| ctg1\_orf11 |  | BGC0000053 | Elaiophylin\_biosynthetic\_gene\_cluster | 32 | 44.2591710338 | 393 | 1.6e-108 |
| AAC38075.1 | polyketide\_synthase\_type\_I | BGC0000127 | Pyoluteorin\_biosynthetic\_gene\_cluster | 31 | 43.2110528823 | 393 | 1.6e-108 |
| ABV97155.1 | Acyl\_transferase | BGC0000137 | Rifamycin\_biosynthetic\_gene\_cluster | 31 | 43.3063363506 | 393 | 2.1e-108 |
| CAQ34929.1 | putative\_polyketide\_synthase | BGC0000986 | Dkxanthene\_biosynthetic\_gene\_cluster | 32 | 43.2586946165 | 393 | 2.1e-108 |
| AEZ53945.1 | polyketide\_synthase | BGC0000144 | Salinomycin\_biosynthetic\_gene\_cluster | 31 | 44.9737970462 | 393 | 2.1e-108 |
| EFL02193.1 | amino\_acid\_adenylation\_domain-containing\_protein | BGC0000996 | Frontalamides\_biosynthetic\_gene\_cluster | 30 | 48.9757027156 | 393 | 2.1e-108 |
| AAZ94387.1 | modular\_polyketide\_synthase | BGC0000040 | Concanamycin\_A\_biosynthetic\_gene\_cluster | 30 | 44.5450214388 | 393 | 2.1e-108 |
| CAP95404.1 |  | BGC0001404 | Sorbicillin\_biosynthetic\_gene\_cluster | 28 | 55.693187232 | 392 | 2.8e-108 |
| ctg1\_orf522 |  | BGC0001199 | Akaeolide\_biosynthetic\_gene\_cluster | 31 | 43.7827536922 | 392 | 2.8e-108 |
| AEK75503.1 | type\_1\_polyketide\_synthase | BGC0000001 | Abyssomicin\_biosynthetic\_gene\_cluster | 30 | 44.9737970462 | 392 | 2.8e-108 |
| AAQ90173.1 | polyketide\_synthase\_type\_I | BGC0000128 | Pyoluteorin\_biosynthetic\_gene\_cluster | 31 | 42.6869938066 | 392 | 3.6e-108 |
| BAV32159.1 | polyketide\_synthase | BGC0001373 | Sordarin\_biosynthetic\_gene\_cluster | 31 | 43.9256788947 | 392 | 3.6e-108 |
| ABJ97439.1 | MerC | BGC0001012 | Meridamycin\_biosynthetic\_gene\_cluster | 30 | 44.9261553121 | 391 | 4.7e-108 |
| ABM21569.1 | CrpA | BGC0000975 | Cryptophycin\_biosynthetic\_gene\_cluster | 30 | 44.7832301096 | 391 | 4.7e-108 |
| ADU85981.1 | putative\_modular\_polyketide\_synthase | BGC0000165 | Tiacumicin\_B\_biosynthetic\_gene\_cluster | 32 | 42.3535016675 | 391 | 8.1e-108 |
| CAD19085.1 | StiA\_protein | BGC0000153 | Stigmatellin\_biosynthetic\_gene\_cluster | 31 | 42.0676512625 | 391 | 8.1e-108 |
| CAE02606.1 | polyketide\_synthase\_type\_I | BGC0000024 | Aureothin\_biosynthetic\_gene\_cluster | 30 | 43.9256788947 | 390 | 1.1e-107 |
| BAO66543.1 | type\_I\_polyketide\_synthase | BGC0000042 | Cremimycin\_biosynthetic\_gene\_cluster | 31 | 43.6398284898 | 390 | 1.1e-107 |
| ACF35446.1 | MbcAII | BGC0000090 | Macbecin\_biosynthetic\_gene\_cluster | 31 | 42.9728442115 | 390 | 1.4e-107 |
| AFL48520.1 | laidlomycin\_polyketide\_synthase\_(module\_9) | BGC0000084 | Laidlomycin\_biosynthetic\_gene\_cluster | 31 | 42.9728442115 | 390 | 1.4e-107 |
| AAO62585.1 | peptide\_sythetase\_polyketide\_synthase\_fusion\_protein | BGC0001016 | Microcystin\_biosynthetic\_gene\_cluster | 29 | 46.6888994759 | 389 | 1.8e-107 |
| AKD43764.1 | HerF | BGC0001349 | Heronamide\_biosynthetic\_gene\_cluster | 31 | 42.1629347308 | 389 | 1.8e-107 |
| ADH04657.1 | TugA | BGC0001342 | Thuggacin\_biosynthetic\_gene\_cluster | 30 | 48.1657932349 | 389 | 2.3e-107 |
| ABB86409.1 | GelB | BGC0000067 | Geldanamycin\_biosynthetic\_gene\_cluster | 28 | 58.07527394 | 389 | 3.1e-107 |
| ADM46357.1 | polyketide\_synthase | BGC0000106 | Naphthomycin\_biosynthetic\_gene\_cluster | 28 | 57.6941400667 | 389 | 3.1e-107 |
| AAK57190.1 | MxaF | BGC0001022 | Myxalamid\_biosynthetic\_gene\_cluster | 31 | 42.0676512625 | 388 | 4e-107 |
| ADC79618.1 | BafAIII | BGC0000028 | Bafilomycin\_biosynthetic\_gene\_cluster | 29 | 48.2610767032 | 388 | 6.8e-107 |
| CAM00065.1 | EryAIII\_Erythromycin\_polyketide\_synthase\_modules\_5\_and\_6 | BGC0000055 | Erythromycin\_biosynthetic\_gene\_cluster | 32 | 42.7346355407 | 388 | 6.8e-107 |
| AEZ53949.1 | polyketide\_synthase | BGC0000144 | Salinomycin\_biosynthetic\_gene\_cluster | 29 | 52.6441162458 | 387 | 8.9e-107 |
| AJW65409.1 | type\_I\_modular\_polyketide\_synthase | BGC0001195 | Nocardiopsin\_biosynthetic\_gene\_cluster | 30 | 43.4492615531 | 387 | 8.9e-107 |
| AAK19883.1 | soraphen\_polyketide\_synthase\_A | BGC0000147 | Soraphen\_biosynthetic\_gene\_cluster | 32 | 42.4487851358 | 387 | 8.9e-107 |
| CQR60494.1 | Polyketide\_synthase,\_type\_I,\_module\_8 | BGC0001287 | Chaxamycin\_A\_/\_chaxamycin\_B\_/\_chaxamycin\_C\_/\_chaxamycin\_D | 31 | 43.8303954264 | 387 | 8.9e-107 |
| AJW65407.1 | type\_I\_modular\_polyketide\_synthase | BGC0001195 | Nocardiopsin\_biosynthetic\_gene\_cluster | 30 | 45.0690805145 | 387 | 8.9e-107 |
| ADC79616.1 | BafAI | BGC0000028 | Bafilomycin\_biosynthetic\_gene\_cluster | 29 | 49.1662696522 | 387 | 8.9e-107 |
| ACO94471.1 | polyketide\_synthase\_type\_I | BGC0000029 | BE-14106\_biosynthetic\_gene\_cluster | 30 | 44.4973797046 | 387 | 1.2e-106 |
| BAK64650.1 | polyketide\_synthase | BGC0000135 | Reveromycin\_biosynthetic\_gene\_cluster | 31 | 44.0686040972 | 386 | 1.5e-106 |
| AAC01713.1 | RifD | BGC0000136 | Rifamycin\_biosynthetic\_gene\_cluster | 32 | 43.0204859457 | 386 | 1.5e-106 |
| CAA60462.1 | polyketide\_synthase | BGC0001040 | Rapamycin\_biosynthetic\_gene\_cluster | 30 | 43.8303954264 | 386 | 1.5e-106 |
| ACY06289.1 | type\_I\_polyketide\_synthase | BGC0001042 | Sanglifehrin\_A\_biosynthetic\_gene\_cluster | 31 | 42.3058599333 | 386 | 1.5e-106 |
| AAO65801.1 | monensin\_polyketide\_synthase\_module\_9 | BGC0000100 | Monensin\_biosynthetic\_gene\_cluster | 30 | 43.7827536922 | 386 | 2e-106 |
| AAC01712.2 | RifC | BGC0000136 | Rifamycin\_biosynthetic\_gene\_cluster | 31 | 43.0204859457 | 386 | 2e-106 |
| BAF85843.1 | modular\_polyketide\_synthase | BGC0000109 | Nemadectin\_biosynthetic\_gene\_cluster | 30 | 44.7832301096 | 386 | 2.6e-106 |
| ABV83222.1 | CppJ | BGC0000116 | Nystatin-like\_Pseudonocardia\_polyene\_biosynthetic\_gene\_cluster | 31 | 45.5931395903 | 386 | 2.6e-106 |
| AAC01711.1 | RifB | BGC0000136 | Rifamycin\_biosynthetic\_gene\_cluster | 29 | 52.4535493092 | 385 | 3.4e-106 |
| AAG13917.1 | megalomicin\_6-deoxyerythronolide\_B\_synthase\_1 | BGC0000092 | Megalomicin\_biosynthetic\_gene\_cluster | 31 | 43.5445450214 | 385 | 3.4e-106 |
| AFL48525.1 | laidlomycin\_polyketide\_synthase\_(loading\_module\_and\_module\_1) | BGC0000084 | Laidlomycin\_biosynthetic\_gene\_cluster | 29 | 42.9252024774 | 385 | 3.4e-106 |
| CAO85897.1 | modular\_polyketide\_synthase\_NorB | BGC0000110 | Neoaureothin\_biosynthetic\_gene\_cluster | 30 | 44.5450214388 | 385 | 3.4e-106 |
| AGI99496.1 | Type\_I\_polyketide\_synthase | BGC0001004 | Lobophorin\_biosynthetic\_gene\_cluster | 31 | 42.4964268699 | 385 | 3.4e-106 |
| BAF85839.1 | modular\_polyketide\_synthase | BGC0000109 | Nemadectin\_biosynthetic\_gene\_cluster | 30 | 43.401619819 | 385 | 4.4e-106 |
| ABC84458.1 | NigAIII | BGC0000114 | Nigericin\_biosynthetic\_gene\_cluster | 29 | 46.2124821343 | 385 | 4.4e-106 |
| ACB46194.1 | polyketide\_synthase | BGC0000989 | Epothilone\_biosynthetic\_gene\_cluster | 29 | 47.8323010958 | 384 | 5.8e-106 |
| AAQ82567.1 | FscE | BGC0000034 | Candicidin\_biosynthetic\_gene\_cluster | 30 | 47.30824202 | 384 | 5.8e-106 |
| AAO65800.1 | monensin\_polyketide\_synthase\_modules\_7\_and\_8 | BGC0000100 | Monensin\_biosynthetic\_gene\_cluster | 30 | 43.8303954264 | 384 | 5.8e-106 |
| ABV83230.1 | CppC | BGC0000116 | Nystatin-like\_Pseudonocardia\_polyene\_biosynthetic\_gene\_cluster | 30 | 49.0233444497 | 384 | 5.8e-106 |
| CAL58682.1 | polyketide\_synthase | BGC0000149 | Spirangien\_biosynthetic\_gene\_cluster | 30 | 43.2110528823 | 384 | 5.8e-106 |
| ABI93779.1 | GdmPKS | BGC0000068 | Geldanamycin\_biosynthetic\_gene\_cluster | 30 | 43.9733206289 | 384 | 7.6e-106 |
| ADU86003.1 | putative\_modular\_polyketide\_synthase | BGC0000165 | Tiacumicin\_B\_biosynthetic\_gene\_cluster | 31 | 43.0204859457 | 384 | 7.6e-106 |
| CAQ52626.1 | type\_I\_polyketide\_synthase,\_loading\_module\_and\_modules\_1-3 | BGC0001066 | Kendomycin\_biosynthetic\_gene\_cluster | 31 | 44.9737970462 | 384 | 7.6e-106 |
| AAG13919.1 | megalomicin\_6-deoxyerythronolide\_B\_synthase\_3 | BGC0000092 | Megalomicin\_biosynthetic\_gene\_cluster | 31 | 44.020962363 | 384 | 9.9e-106 |
| AHB82062.1 | polyketide\_synthase | BGC0001231 | Microsclerodermins\_biosynthetic\_gene\_cluster | 30 | 44.020962363 | 384 | 9.9e-106 |
| CQR60496.1 | Polyketide\_synthase,\_type\_I,\_modules:\_4,\_5\_and\_6 | BGC0001287 | Chaxamycin\_A\_/\_chaxamycin\_B\_/\_chaxamycin\_C\_/\_chaxamycin\_D | 29 | 47.3558837542 | 384 | 9.9e-106 |
| AGC24271.1 | PrlQ | BGC0001038 | Pyralomicin\_biosynthetic\_gene\_cluster | 31 | 42.6393520724 | 383 | 1.3e-105 |
| CAJ88186.1 | putative\_modular\_polyketide\_synthase | BGC0000151 | Stambomycin\_biosynthetic\_gene\_cluster | 31 | 42.9252024774 | 383 | 1.3e-105 |
| CBA11584.1 | polyketide\_synthase\_type\_I | BGC0001046 | Streptolydigin\_biosynthetic\_gene\_cluster | 31 | 44.2591710338 | 383 | 1.7e-105 |
| ABC84469.1 | NigAIX | BGC0000114 | Nigericin\_biosynthetic\_gene\_cluster | 30 | 47.6417341591 | 383 | 1.7e-105 |
| ABC84456.1 | NigAI | BGC0000114 | Nigericin\_biosynthetic\_gene\_cluster | 30 | 44.0686040972 | 383 | 1.7e-105 |
| AAZ77699.1 | ChlA6 | BGC0000036 | Chlorothricin\_biosynthetic\_gene\_cluster | 31 | 43.4492615531 | 383 | 1.7e-105 |
| AJW65408.1 | type\_I\_modular\_polyketide\_synthase | BGC0001195 | Nocardiopsin\_biosynthetic\_gene\_cluster | 31 | 44.0686040972 | 383 | 2.2e-105 |
| AFV30250.1 | polyketide\_synthase | BGC0000075 | Hygrocin\_biosynthetic\_gene\_cluster | 30 | 43.5445450214 | 382 | 2.9e-105 |
| ADC45586.1 | modular\_polyketide\_synthase | BGC0000093 | Meilingmycin\_biosynthetic\_gene\_cluster | 29 | 49.6426869938 | 382 | 2.9e-105 |
| AAX98185.1 | polyketide\_synthase\_type\_I | BGC0000052 | ECO-02301\_biosynthetic\_gene\_cluster | 31 | 43.3063363506 | 382 | 3.7e-105 |
| CAE02605.1 | polyketide\_synthase\_type\_I | BGC0000024 | Aureothin\_biosynthetic\_gene\_cluster | 31 | 43.5921867556 | 382 | 3.7e-105 |
| ACO94470.1 | polyketide\_synthase\_type\_I | BGC0000029 | BE-14106\_biosynthetic\_gene\_cluster | 30 | 43.2110528823 | 381 | 4.9e-105 |
| AHH99922.1 | PKS\_I | BGC0000002 | Aculeximycin\_biosynthetic\_gene\_cluster | 30 | 44.2115292997 | 381 | 4.9e-105 |
| ACF35445.1 | MbcAI | BGC0000090 | Macbecin\_biosynthetic\_gene\_cluster | 31 | 43.3063363506 | 381 | 4.9e-105 |
| AAZ94386.1 | modular\_polyketide\_synthase | BGC0000040 | Concanamycin\_A\_biosynthetic\_gene\_cluster | 32 | 43.7351119581 | 381 | 4.9e-105 |
| CAI94682.1 | putative\_polyketide\_synthase | BGC0000141 | Rubradirin\_biosynthetic\_gene\_cluster | 30 | 43.9256788947 | 381 | 4.9e-105 |
| AAF71768.1 | NysK | BGC0000115 | Nystatin\_biosynthetic\_gene\_cluster | 31 | 43.1634111482 | 381 | 6.4e-105 |
| AHB82072.1 | non\_ribosomal\_peptide\_synthetase/polyketide\_synthase | BGC0001231 | Microsclerodermins\_biosynthetic\_gene\_cluster | 31 | 43.5445450214 | 381 | 6.4e-105 |
| EAU29529.1 | hypothetical\_protein | BGC0000682 | Terretonin\_biosynthetic\_gene\_cluster | 27 | 67.2224868985 | 381 | 6.4e-105 |
| CAA60459.1 | polyketide\_synthase | BGC0001040 | Rapamycin\_biosynthetic\_gene\_cluster | 30 | 42.4011434016 | 381 | 6.4e-105 |
| ABV97152.1 | Beta-ketoacyl\_synthase | BGC0000137 | Rifamycin\_biosynthetic\_gene\_cluster | 30 | 46.7365412101 | 381 | 8.4e-105 |
| AEC13069.1 | fosC | BGC0000060 | Fostriecin\_biosynthetic\_gene\_cluster | 31 | 43.7351119581 | 381 | 8.4e-105 |
| ABC84459.1 | NigAIV | BGC0000114 | Nigericin\_biosynthetic\_gene\_cluster | 31 | 45.5931395903 | 380 | 1.1e-104 |
| AAG23264.1 | polyketide\_synthase\_loading\_and\_extender\_module\_1 | BGC0000148 | Spinosad\_biosynthetic\_gene\_cluster | 31 | 43.3063363506 | 380 | 1.1e-104 |
| CAE45670.1 | borrelidin\_polyketide\_synthase,\_type\_I | BGC0000031 | Borrelidin\_biosynthetic\_gene\_cluster | 30 | 42.5440686041 | 380 | 1.1e-104 |
| CAA60460.1 | polyketide\_synthase | BGC0001040 | Rapamycin\_biosynthetic\_gene\_cluster | 30 | 43.4969032873 | 380 | 1.4e-104 |
| ACY06287.1 | type\_I\_polyketide\_synthase | BGC0001042 | Sanglifehrin\_A\_biosynthetic\_gene\_cluster | 32 | 43.5921867556 | 379 | 1.9e-104 |
| AAY28226.1 | HbmAII | BGC0000074 | Herbimycin\_biosynthetic\_gene\_cluster | 28 | 58.2658408766 | 379 | 1.9e-104 |
| ACB46471.1 | polyketide\_synthase | BGC0000082 | Kijanimicin\_biosynthetic\_gene\_cluster | 30 | 43.8780371606 | 379 | 1.9e-104 |
| CAM00064.1 | EryAII\_Erythromycin\_polyketide\_synthase\_modules\_3\_and\_4 | BGC0000055 | Erythromycin\_biosynthetic\_gene\_cluster | 29 | 49.8332539304 | 379 | 2.4e-104 |
| ACO94498.1 | polyketide\_synthase\_type\_I | BGC0000097 | ML-449\_biosynthetic\_gene\_cluster | 30 | 42.9728442115 | 379 | 3.2e-104 |
| ADA69241.1 | cis-AT\_polyketide\_synthase | BGC0001071 | Nosperin\_biosynthetic\_gene\_cluster | 31 | 43.2586946165 | 379 | 3.2e-104 |
| BAJ16471.1 | polyketide\_synthase | BGC0000058 | FD-891\_biosynthetic\_gene\_cluster | 30 | 47.4988089566 | 378 | 4.1e-104 |
| BAQ25507.1 | type\_I\_polyketide\_synthase | BGC0001288 | Maklamicin\_biosynthetic\_gene\_cluster | 32 | 42.6869938066 | 378 | 4.1e-104 |
| EAQ86392.1 | hypothetical\_protein | BGC0001405 | Chaetoviridin\_/\_chaetomugilin\_biosynthetic\_gene\_cluster | 32 | 37.3034778466 | 378 | 4.1e-104 |
| AAM70355.1 | CalO5 | BGC0000033 | Calicheamicin\_biosynthetic\_gene\_cluster | 30 | 46.9747498809 | 378 | 4.1e-104 |
| BAK64649.1 | polyketide\_synthase | BGC0000135 | Reveromycin\_biosynthetic\_gene\_cluster | 30 | 44.020962363 | 378 | 5.4e-104 |
| ADZ24995.1 | non-ribosomal\_peptide\_synthase/polyketide\_synthase | BGC0000380 | Leupyrrin\_biosynthetic\_gene\_cluster | 30 | 48.2610767032 | 378 | 5.4e-104 |
| CAJ88184.1 | putative\_modular\_polyketide\_synthase | BGC0000151 | Stambomycin\_biosynthetic\_gene\_cluster | 30 | 42.8299190091 | 378 | 5.4e-104 |
| CBD77746.1 | non-ribosomal\_peptide\_synthetase/polyketide\_synthase | BGC0000974 | Crocacin\_biosynthetic\_gene\_cluster | 30 | 43.6398284898 | 378 | 7.1e-104 |
| AGI99482.1 | Type\_I\_polyketide\_synthase | BGC0001004 | Lobophorin\_biosynthetic\_gene\_cluster | 30 | 44.8308718437 | 377 | 9.2e-104 |
| AGC09499.1 | LobS4 | BGC0001183 | Lobophorin\_biosynthetic\_gene\_cluster | 30 | 44.8308718437 | 377 | 9.2e-104 |
| CAJ88177.1 | putative\_type\_I\_polyketide\_synthase | BGC0000151 | Stambomycin\_biosynthetic\_gene\_cluster | 30 | 42.4964268699 | 377 | 1.2e-103 |
| BAP34733.1 | type\_I\_polyketide\_synthase | BGC0000078 | Incednine\_biosynthetic\_gene\_cluster | 28 | 48.8327775131 | 376 | 1.6e-103 |
| ADC45515.1 | modular\_polyketide\_synthase | BGC0000093 | Meilingmycin\_biosynthetic\_gene\_cluster | 31 | 42.7822772749 | 376 | 1.6e-103 |
| AHB82070.1 | polyketide\_synthase | BGC0001231 | Microsclerodermins\_biosynthetic\_gene\_cluster | 30 | 44.5926631729 | 376 | 2.7e-103 |
| AHH99923.1 | PKS\_I | BGC0000002 | Aculeximycin\_biosynthetic\_gene\_cluster | 31 | 43.5445450214 | 376 | 2.7e-103 |
| CAD55506.1 | putative\_type\_I\_polyketide\_synthase | BGC0000038 | Coelimycin\_biosynthetic\_gene\_cluster | 29 | 47.7846593616 | 376 | 2.7e-103 |
| CAF05649.1 | TubD\_protein | BGC0001053 | Tubulysin\_biosynthetic\_gene\_cluster | 31 | 43.0681276798 | 375 | 3.5e-103 |
| AAP42859.1 | NanA5 | BGC0000105 | Nanchangmycin\_biosynthetic\_gene\_cluster | 31 | 44.6879466413 | 375 | 3.5e-103 |
| ABV83229.1 | CppB | BGC0000116 | Nystatin-like\_Pseudonocardia\_polyene\_biosynthetic\_gene\_cluster | 30 | 43.3063363506 | 375 | 4.6e-103 |
| CAI94713.1 | putative\_polyketide\_synthase | BGC0000141 | Rubradirin\_biosynthetic\_gene\_cluster | 29 | 48.2610767032 | 375 | 4.6e-103 |
| ABP55222.1 | beta-ketoacyl\_synthase | BGC0000142 | Salinilactam\_biosynthetic\_gene\_cluster | 30 | 43.4492615531 | 375 | 4.6e-103 |
| ADC79637.1 | TamAI | BGC0001052 | Tirandamycin\_biosynthetic\_gene\_cluster | 30 | 43.0204859457 | 374 | 6e-103 |
| BAB69192.1 | modular\_polyketide\_synthase | BGC0000117 | Oligomycin\_biosynthetic\_gene\_cluster | 30 | 46.5936160076 | 374 | 7.8e-103 |
| ABW96542.1 | type\_I\_modular\_polyketide\_synthase | BGC0000159 | Tautomycin\_biosynthetic\_gene\_cluster | 30 | 43.4492615531 | 374 | 7.8e-103 |
| AAP42855.1 | NanA1 | BGC0000105 | Nanchangmycin\_biosynthetic\_gene\_cluster | 30 | 44.2115292997 | 374 | 7.8e-103 |
| CAD29795.1 | peptide\_synthetase | BGC0001015 | Microcystin\_biosynthetic\_gene\_cluster | 29 | 43.8303954264 | 374 | 1e-102 |
| ctg1\_orf10 |  | BGC0000053 | Elaiophylin\_biosynthetic\_gene\_cluster | 29 | 44.8785135779 | 373 | 1.3e-102 |
| ABV97153.1 | Beta-ketoacyl\_synthase | BGC0000137 | Rifamycin\_biosynthetic\_gene\_cluster | 31 | 44.7355883754 | 373 | 2.3e-102 |
| CAQ18832.1 | polyketide\_synthase | BGC0000954 | Ajudazol\_biosynthetic\_gene\_cluster | 28 | 58.4087660791 | 372 | 3e-102 |
| AAU93807.2 | polyketide\_synthase\_modules\_1\_and\_2 | BGC0000054 | Erythromycin\_biosynthetic\_gene\_cluster | 30 | 42.8775607432 | 372 | 3e-102 |
| ctg1\_orf8 |  | BGC0001109 | Pederin\_biosynthetic\_gene\_cluster | 33 | 25.3930443068 | 281 | 9e-75 |
| AHH99926.1 | PKS\_I | BGC0000002 | Aculeximycin\_biosynthetic\_gene\_cluster | 31 | 42.7822772749 | 372 | 3.9e-102 |
| AAF00957.1 | McyG | BGC0001017 | Microcystin\_biosynthetic\_gene\_cluster | 30 | 43.4969032873 | 372 | 3.9e-102 |
| AJO72736.1 | Type\_I\_modular\_polyketide\_synthase | BGC0001381 | Brasilinolide\_biosynthetic\_gene\_cluster | 31 | 43.7827536922 | 372 | 3.9e-102 |
| ABV91286.1 | type\_I\_modular\_polyketide\_synthase | BGC0000158 | Tautomycetin\_biosynthetic\_gene\_cluster | 31 | 42.8299190091 | 372 | 3.9e-102 |
| CAF05651.1 | TubF\_protein | BGC0001053 | Tubulysin\_biosynthetic\_gene\_cluster | 31 | 43.0681276798 | 372 | 3.9e-102 |
| AAX98190.1 | polyketide\_synthase\_type\_I | BGC0000052 | ECO-02301\_biosynthetic\_gene\_cluster | 30 | 43.5445450214 | 371 | 5.1e-102 |
| AEZ53950.1 | polyketide\_synthase | BGC0000144 | Salinomycin\_biosynthetic\_gene\_cluster | 26 | 66.6031443545 | 371 | 5.1e-102 |
| AFL48532.1 | laidlomycin\_polyketide\_synthase\_(module\_11\_and\_module\_12) | BGC0000084 | Laidlomycin\_biosynthetic\_gene\_cluster | 30 | 45.2596474512 | 371 | 6.6e-102 |
| ABI94380.1 | tautomycetin\_biosynthetic\_PKS | BGC0000157 | Tautomycetin\_biosynthetic\_gene\_cluster | 30 | 43.2586946165 | 371 | 8.6e-102 |
| CAJ88185.2 | putative\_modular\_polyketide\_synthase | BGC0000151 | Stambomycin\_biosynthetic\_gene\_cluster | 29 | 43.2110528823 | 371 | 8.6e-102 |
| AAQ84156.1 | Plm1 | BGC0000123 | Phoslactomycin\_B\_biosynthetic\_gene\_cluster | 30 | 44.020962363 | 371 | 8.6e-102 |
| AKD43753.1 | HerB | BGC0001349 | Heronamide\_biosynthetic\_gene\_cluster | 29 | 47.30824202 | 371 | 8.6e-102 |
| ABG02264.1 | SalB | BGC0000143 | Salinomycin\_biosynthetic\_gene\_cluster | 31 | 43.1634111482 | 370 | 1.5e-101 |
| BAE93731.1 | type\_I\_polyketide\_synthase | BGC0000164 | Tetronomycin\_biosynthetic\_gene\_cluster | 30 | 47.4035254883 | 370 | 1.5e-101 |
| AAC46026.1 | polyketide\_synthase\_modules\_4\_and\_5 | BGC0000113 | Niddamycin\_biosynthetic\_gene\_cluster | 30 | 44.5926631729 | 369 | 1.9e-101 |
| CBZ41586.1 | putative\_modular\_polyketide\_synthase | BGC0000151 | Stambomycin\_biosynthetic\_gene\_cluster | 31 | 42.4964268699 | 369 | 1.9e-101 |
| ADH04680.1 | hybrid\_polyketide\_synthase/non-ribosomal\_peptide\_synthetase | BGC0001344 | Tubulysin\_biosynthetic\_gene\_cluster | 31 | 44.2115292997 | 369 | 1.9e-101 |
| AHD05614.1 | putative\_non-ribosomal\_peptide\_ligase/\_polyketide\_synthase\_hybrid | BGC0001033 | Paenilamicin\_biosynthetic\_gene\_cluster | 31 | 43.2110528823 | 369 | 1.9e-101 |
| CBA11583.1 | polyketide\_synthase\_type\_I | BGC0001046 | Streptolydigin\_biosynthetic\_gene\_cluster | 30 | 44.5926631729 | 369 | 1.9e-101 |
| ABI94379.1 | tautomycetin\_biosynthetic\_PKS | BGC0000157 | Tautomycetin\_biosynthetic\_gene\_cluster | 31 | 42.8299190091 | 369 | 1.9e-101 |
| ABB88519.1 | polyketide\_synthase\_type\_I | BGC0000050 | E-837\_biosynthetic\_gene\_cluster | 31 | 45.7837065269 | 369 | 1.9e-101 |
| ABC87509.1 | polyketide\_synthase | BGC0001011 | Meridamycin\_biosynthetic\_gene\_cluster | 30 | 44.7355883754 | 369 | 2.5e-101 |
| ctg1\_orf20 |  | BGC0001013 | Meridamycin\_biosynthetic\_gene\_cluster | 30 | 44.7355883754 | 369 | 2.5e-101 |
| BAC76491.1 | lankamycin\_synthase\_LkmAIII | BGC0000085 | Lankamycin\_biosynthetic\_gene\_cluster | 31 | 43.3539780848 | 369 | 2.5e-101 |
| AAP85336.1 | type\_I\_PKS | BGC0000233 | Hedamycin\_biosynthetic\_gene\_cluster | 31 | 43.2586946165 | 369 | 2.5e-101 |
| CAQ52623.1 | type\_I\_polyketide\_synthase,\_module\_6 | BGC0001066 | Kendomycin\_biosynthetic\_gene\_cluster | 29 | 43.401619819 | 369 | 3.3e-101 |
| KGO40478.1 | Acyl\_transferase/acyl\_hydrolase/lysophospholipase | BGC0001205 | Communesin\_biosynthetic\_gene\_cluster | 30 | 45.3549309195 | 369 | 3.3e-101 |
| ACB37741.1 | putative\_type\_I\_polyketide\_synthase | BGC0000162 | Tetrocarcin\_A\_biosynthetic\_gene\_cluster | 30 | 45.4502143878 | 369 | 3.3e-101 |
| AAD03048.1 | type\_I\_polyketide\_synthase | BGC0000041 | Coronafacic\_acid\_biosynthetic\_gene\_cluster | 29 | 45.7837065269 | 368 | 4.3e-101 |
| ACN69992.1 | polyketide\_synthase | BGC0000079 | Indanomycin\_biosynthetic\_gene\_cluster | 29 | 44.306812768 | 368 | 4.3e-101 |
| AEZ53953.1 | polyketide\_synthase | BGC0000144 | Salinomycin\_biosynthetic\_gene\_cluster | 29 | 45.2596474512 | 368 | 4.3e-101 |
| AAR87760.2 | ZmaK | BGC0001059 | Zwittermycin\_A\_biosynthetic\_gene\_cluster | 31 | 43.0204859457 | 368 | 4.3e-101 |
| AGY62759.1 | EbeG | BGC0000051 | Ebelactone\_biosynthetic\_gene\_cluster | 30 | 43.4969032873 | 368 | 4.3e-101 |
| AHA38202.1 | GphI | BGC0000069 | Gephyronic\_acid\_biosynthetic\_gene\_cluster | 30 | 48.3087184374 | 368 | 5.6e-101 |
| AIT55264.1 | GulA | BGC0000072 | Gulmirecin\_biosynthetic\_gene\_cluster | 30 | 42.1629347308 | 368 | 5.6e-101 |
| AKD43761.1 | HerD | BGC0001349 | Heronamide\_biosynthetic\_gene\_cluster | 30 | 42.210576465 | 368 | 5.6e-101 |
| BAP34763.1 | type\_I\_polyketide\_synthase | BGC0000078 | Incednine\_biosynthetic\_gene\_cluster | 29 | 49.7379704621 | 368 | 7.3e-101 |
| ACO94499.1 | polyketide\_synthase\_type\_I | BGC0000097 | ML-449\_biosynthetic\_gene\_cluster | 30 | 43.5445450214 | 367 | 9.6e-101 |
| AEU17897.1 | putative\_type\_I\_PKS | BGC0001072 | Simocyclinone\_biosynthetic\_gene\_cluster | 29 | 48.0228680324 | 367 | 9.6e-101 |
| ABV97151.1 | AMP-dependent\_synthetase\_and\_ligase | BGC0000137 | Rifamycin\_biosynthetic\_gene\_cluster | 31 | 43.3063363506 | 367 | 1.2e-100 |
| AAY28225.1 | HbmAI | BGC0000074 | Herbimycin\_biosynthetic\_gene\_cluster | 29 | 47.3558837542 | 366 | 1.6e-100 |
| EAA65602.1 | hypothetical\_protein | BGC0000022 | Asperfuranone\_biosynthetic\_gene\_cluster | 30 | 44.4497379705 | 366 | 1.6e-100 |
| IF55\_RS36525 | polyketide\_synthase | BGC0001348 | JBIR-100\_biosynthetic\_gene\_cluster | 30 | 43.7351119581 | 366 | 1.6e-100 |
| AAZ94390.1 | modular\_polyketide\_synthase | BGC0000040 | Concanamycin\_A\_biosynthetic\_gene\_cluster | 30 | 44.1162458313 | 366 | 1.6e-100 |
| AAD34559.1 | polyketide\_synthase | BGC0000089 | Lovastatin\_biosynthetic\_gene\_cluster | 33 | 37.6369699857 | 366 | 2.1e-100 |
| ADC79638.1 | TamAII | BGC0001052 | Tirandamycin\_biosynthetic\_gene\_cluster | 29 | 44.2591710338 | 366 | 2.1e-100 |
| AHD05619.1 | putative\_polyketide\_synthase\_subunit | BGC0001033 | Paenilamicin\_biosynthetic\_gene\_cluster | 30 | 43.8780371606 | 366 | 2.8e-100 |
| BAB69195.1 | modular\_polyketide\_synthase | BGC0000117 | Oligomycin\_biosynthetic\_gene\_cluster | 30 | 43.9256788947 | 364 | 8.1e-100 |
| CAL58681.1 | polyketide\_synthase | BGC0000149 | Spirangien\_biosynthetic\_gene\_cluster | 31 | 41.7341591234 | 364 | 8.1e-100 |
| ABC84460.1 | NigAV | BGC0000114 | Nigericin\_biosynthetic\_gene\_cluster | 30 | 43.7827536922 | 364 | 1.1e-99 |
| ALA09371.1 | type\_I\_modular\_PKS | BGC0001303 | Lobosamide\_biosynthetic\_gene\_cluster | 29 | 49.0709861839 | 364 | 1.1e-99 |
| ABV91287.1 | type\_I\_modular\_polyketide\_synthase | BGC0000158 | Tautomycetin\_biosynthetic\_gene\_cluster | 30 | 43.3539780848 | 364 | 1.1e-99 |
| BAC57028.1 | protomycinolide\_IV\_synthase\_1 | BGC0000102 | Mycinamicin\_biosynthetic\_gene\_cluster | 29 | 45.0214387804 | 363 | 1.4e-99 |
| AAP42874.1 | NanA8 | BGC0000105 | Nanchangmycin\_biosynthetic\_gene\_cluster | 30 | 43.7827536922 | 363 | 1.4e-99 |
| CAQ18838.1 | polyketide\_synthase | BGC0000954 | Ajudazol\_biosynthetic\_gene\_cluster | 30 | 41.4006669843 | 363 | 1.4e-99 |
| AAD03047.1 | type\_I\_polyketide\_synthase | BGC0000041 | Coronafacic\_acid\_biosynthetic\_gene\_cluster | 29 | 42.7346355407 | 363 | 1.8e-99 |
| CAJ88187.2 | putative\_type\_I\_modular\_polyketide\_synthase | BGC0000151 | Stambomycin\_biosynthetic\_gene\_cluster | 30 | 42.9252024774 | 363 | 2.4e-99 |
| ABC87510.1 | polyketide\_synthase | BGC0001011 | Meridamycin\_biosynthetic\_gene\_cluster | 30 | 43.3539780848 | 362 | 3.1e-99 |
| ctg1\_orf21 |  | BGC0001013 | Meridamycin\_biosynthetic\_gene\_cluster | 30 | 43.3539780848 | 362 | 3.1e-99 |
| AHA38200.1 | GphG | BGC0000069 | Gephyronic\_acid\_biosynthetic\_gene\_cluster | 33 | 39.3997141496 | 362 | 3.1e-99 |
| AEZ53948.1 | polyketide\_synthase | BGC0000144 | Salinomycin\_biosynthetic\_gene\_cluster | 29 | 43.5445450214 | 362 | 4e-99 |
| WP\_043476519.1 | polyketide\_synthase | BGC0001232 | Leucanicidin\_biosynthetic\_gene\_cluster | 30 | 44.1162458313 | 361 | 6.8e-99 |
| ADC45516.1 | modular\_polyketide\_synthase | BGC0000093 | Meilingmycin\_biosynthetic\_gene\_cluster | 30 | 42.6393520724 | 361 | 6.8e-99 |
| CAO98852.1 | polyketide\_synthase\_AufI | BGC0000023 | Aurafuron\_biosynthetic\_gene\_cluster | 31 | 41.4959504526 | 361 | 6.8e-99 |
| CAE14173.1 |  | BGC0000383 | Luminmycin\_biosynthetic\_gene\_cluster | 30 | 45.4025726536 | 361 | 6.8e-99 |
| BAC76492.1 | lankamycin\_synthase\_LkmAII | BGC0000085 | Lankamycin\_biosynthetic\_gene\_cluster | 30 | 43.3539780848 | 359 | 2.6e-98 |
| AAS79461.1 | polyketide\_synthase\_subunit | BGC0000035 | Chalcomycin\_biosynthetic\_gene\_cluster | 29 | 45.7360647928 | 359 | 3.4e-98 |
| CCE88377.1 | non-ribosomal\_peptide\_synthetase/polyketide\_synthase | BGC0001034 | Pellasoren\_biosynthetic\_gene\_cluster | 30 | 42.6393520724 | 358 | 4.4e-98 |
| CAO85893.1 | modular\_polyketide\_synthase\_NorA | BGC0000110 | Neoaureothin\_biosynthetic\_gene\_cluster | 30 | 42.9252024774 | 358 | 4.4e-98 |
| ADH04682.1 | polyketide\_synthase | BGC0001344 | Tubulysin\_biosynthetic\_gene\_cluster | 30 | 43.1634111482 | 358 | 4.4e-98 |
| ctg1\_orf9 |  | BGC0000053 | Elaiophylin\_biosynthetic\_gene\_cluster | 29 | 43.4492615531 | 358 | 7.6e-98 |
| ctg1\_orf0002 |  | BGC0001068 | Pyripyropene\_A\_biosynthetic\_gene\_cluster | 30 | 44.3544545021 | 357 | 9.9e-98 |
| AKL78824.1 | GLPKS3 | BGC0001187 | Xenolozoyenone\_biosynthetic\_gene\_cluster | 27 | 51.786565031 | 357 | 1.3e-97 |
| AAP42860.1 | NanA6 | BGC0000105 | Nanchangmycin\_biosynthetic\_gene\_cluster | 30 | 43.3539780848 | 357 | 1.3e-97 |
| AAY89049.1 | polyketide\_synthase | BGC0001069 | Chivosazole\_biosynthetic\_gene\_cluster | 29 | 42.1629347308 | 356 | 1.7e-97 |
| BAE93730.1 | type\_I\_polyketide\_synthase | BGC0000164 | Tetronomycin\_biosynthetic\_gene\_cluster | 29 | 47.7370176274 | 356 | 2.2e-97 |
| CAJ76291.1 | putative\_polyketide\_synthase | BGC0000972 | Colibactin\_biosynthetic\_gene\_cluster | 29 | 43.6874702239 | 356 | 2.2e-97 |
| AAU93806.2 | polyketide\_synthase\_modules\_3\_and\_4 | BGC0000054 | Erythromycin\_biosynthetic\_gene\_cluster | 30 | 43.0204859457 | 354 | 6.4e-97 |
| AAF86393.1 | FkbB | BGC0000994 | FK520\_biosynthetic\_gene\_cluster | 29 | 46.7841829443 | 354 | 8.4e-97 |
| BAC57031.1 | protomycinolide\_IV\_synthase\_4 | BGC0000102 | Mycinamicin\_biosynthetic\_gene\_cluster | 30 | 44.3544545021 | 354 | 1.1e-96 |
| ACF35447.1 | MbcAIII | BGC0000090 | Macbecin\_biosynthetic\_gene\_cluster | 30 | 43.8303954264 | 354 | 1.1e-96 |
| AAM77986.1 | iterative\_type\_I\_polyketide\_synthase | BGC0000112 | Neocarzinostatin\_biosynthetic\_gene\_cluster | 29 | 43.4492615531 | 353 | 1.4e-96 |
| ABA02240.1 | polyketide\_synthase | BGC0000098 | Monacolin\_K\_biosynthetic\_gene\_cluster | 33 | 36.6364935684 | 353 | 1.9e-96 |
| ctg1\_orf30 |  | BGC0000096 | Midecamycin\_biosynthetic\_gene\_cluster | 30 | 43.6398284898 | 352 | 2.4e-96 |
| ABJ97438.1 | MerB | BGC0001012 | Meridamycin\_biosynthetic\_gene\_cluster | 30 | 43.1634111482 | 352 | 2.4e-96 |
| BAD38873.1 | polyketide\_synthase | BGC0000111 | Neocarzilin\_biosynthetic\_gene\_cluster | 30 | 43.7827536922 | 351 | 9.3e-96 |
| ABL74938.1 | TlmVIII | BGC0001048 | Tallysomycin\_biosynthetic\_gene\_cluster | 30 | 43.4492615531 | 350 | 1.2e-95 |
| EHA28244.1 | hypothetical\_protein | BGC0001143 | Azanigerone\_biosynthetic\_gene\_cluster | 30 | 44.8308718437 | 350 | 1.2e-95 |
| EAL89230.2 | LovB-like\_polyketide\_synthase,\_putative | BGC0000129 | Pyripyropene\_biosynthetic\_gene\_cluster | 30 | 44.5450214388 | 350 | 1.6e-95 |
| ABF87031.1 | non-ribosomal\_peptide\_synthetase/polyketide\_synthase | BGC0000393 | Myxoprincomide\_biosynthetic\_gene\_cluster | 29 | 43.0681276798 | 349 | 3.5e-95 |
| AHB82057.1 | polyketide\_synthase | BGC0001019 | Microsclerodermins\_biosynthetic\_gene\_cluster | 29 | 43.5445450214 | 348 | 4.6e-95 |
| AHB82065.1 | polyketide\_synthase | BGC0001231 | Microsclerodermins\_biosynthetic\_gene\_cluster | 35 | 31.4911862792 | 348 | 4.6e-95 |
| BAD97694.1 | Aft9-1 | BGC0000003 | AF-toxin\_biosynthetic\_gene\_cluster | 33 | 39.6379228204 | 347 | 7.8e-95 |
| AAF86396.1 | FkbA | BGC0000994 | FK520\_biosynthetic\_gene\_cluster | 29 | 42.3058599333 | 347 | 7.8e-95 |
| AAO06916.1 | GdmAI | BGC0000066 | Geldanamycin\_biosynthetic\_gene\_cluster | 28 | 48.1657932349 | 347 | 7.8e-95 |
| CCM44338.1 | putative\_polyketide\_synthase | BGC0001056 | Zeamine\_biosynthetic\_gene\_cluster | 29 | 43.1634111482 | 347 | 1e-94 |
| ACD39767.1 | reducing\_polyketide\_synthase | BGC0000077 | Hypothemycin\_biosynthetic\_gene\_cluster | 31 | 40.0666984278 | 347 | 1.3e-94 |
| ACD39758.1 | reducing\_polyketide\_synthase | BGC0000076 | Hypothemycin\_biosynthetic\_gene\_cluster | 31 | 40.0666984278 | 347 | 1.3e-94 |
| BAC20566.1 | polyketide\_synthase | BGC0000039 | Compactin\_biosynthetic\_gene\_cluster | 33 | 36.4935683659 | 346 | 2.3e-94 |
| AAZ94389.1 | modular\_polyketide\_synthase | BGC0000040 | Concanamycin\_A\_biosynthetic\_gene\_cluster | 29 | 41.8294425917 | 346 | 3e-94 |
| AHB82063.1 | polyketide\_synthase | BGC0001231 | Microsclerodermins\_biosynthetic\_gene\_cluster | 35 | 32.634587899 | 345 | 3.9e-94 |
| CCC55921.1 | non-ribosomal\_peptide\_synthetase/polyketide\_synthase\_hybrid\_protein | BGC0000973 | Collismycin\_A\_biosynthetic\_gene\_cluster | 30 | 42.9728442115 | 345 | 5.1e-94 |
| CAP95405.1 |  | BGC0001404 | Sorbicillin\_biosynthetic\_gene\_cluster | 29 | 41.305383516 | 345 | 5.1e-94 |
| ABY83164.1 | Azi26 | BGC0000960 | Azinomycin\_B\_biosynthetic\_gene\_cluster | 29 | 43.5445450214 | 344 | 8.7e-94 |
| CCA29203.1 | non-ribosomal\_peptide\_synthetase/polyketide\_synthase | BGC0000955 | Althiomycin\_biosynthetic\_gene\_cluster | 29 | 43.0681276798 | 344 | 1.1e-93 |
| AAB08104.3 | polyketide\_synthase | BGC0000155\_c3 | T-toxin\_biosynthetic\_gene\_cluster | 30 | 45.1643639828 | 344 | 1.1e-93 |
| ABB86408.1 | GelA | BGC0000067 | Geldanamycin\_biosynthetic\_gene\_cluster | 28 | 48.1657932349 | 344 | 1.1e-93 |
| AJO72742.1 | Type\_I\_modular\_polyketide\_synthase | BGC0001381 | Brasilinolide\_biosynthetic\_gene\_cluster | 30 | 40.4954740353 | 343 | 1.5e-93 |
| AHD05615.1 | putative\_non-ribosomal\_peptide\_ligase/\_polyketide\_synthase\_hybrid | BGC0001033 | Paenilamicin\_biosynthetic\_gene\_cluster | 30 | 42.9252024774 | 343 | 1.5e-93 |
| ABX37384.1 | Beta-ketoacyl\_synthase | BGC0000984 | Delftibactin\_biosynthetic\_gene\_cluster | 29 | 44.2115292997 | 342 | 2.5e-93 |
| CAL80821.1 | sylD-like\_NRPS/PKS | BGC0000997 | Glidobactin\_biosynthetic\_gene\_cluster | 30 | 43.1634111482 | 339 | 2.1e-92 |
| AHB82054.1 | polyketide\_synthase | BGC0001019 | Microsclerodermins\_biosynthetic\_gene\_cluster | 34 | 31.0624106717 | 339 | 2.1e-92 |
| AAU93805.2 | polyketide\_synthase\_modules\_5\_and\_6 | BGC0000054 | Erythromycin\_biosynthetic\_gene\_cluster | 30 | 44.4973797046 | 339 | 2.8e-92 |
| AAO65796.1 | monensin\_polyketide\_synthase\_loading\_module\_and\_module\_1 | BGC0000100 | Monensin\_biosynthetic\_gene\_cluster | 31 | 44.6879466413 | 339 | 3.6e-92 |
| ABB90283.1 | polyketide\_synthase | BGC0001057 | Zearalenone\_biosynthetic\_gene\_cluster | 30 | 43.7827536922 | 338 | 4.8e-92 |
| EJK79843.1 | amino\_acid\_adenylation\_enzyme/thioester\_reductase\_family\_protein | BGC0000436 | Syringolin\_A\_biosynthetic\_gene\_cluster | 29 | 44.5450214388 | 337 | 1.1e-91 |
| AFD30954.1 | CrmA | BGC0000966 | Caerulomycin\_A\_biosynthetic\_gene\_cluster | 29 | 44.0686040972 | 336 | 1.8e-91 |
| AGY62753.1 | EbeA | BGC0000051 | Ebelactone\_biosynthetic\_gene\_cluster | 30 | 39.5902810862 | 336 | 3.1e-91 |
| ABB76806.1 | polyketide\_synthase\_2 | BGC0000155\_c2 | T-toxin\_biosynthetic\_gene\_cluster | 30 | 40.5907575036 | 333 | 1.5e-90 |
| AHB82052.1 | polyketide\_synthase | BGC0001019 | Microsclerodermins\_biosynthetic\_gene\_cluster | 33 | 32.634587899 | 333 | 1.5e-90 |
| AAF86392.1 | FkbC | BGC0000994 | FK520\_biosynthetic\_gene\_cluster | 28 | 48.4516436398 | 331 | 7.6e-90 |
| AKA54627.1 | PKS | BGC0001216 | Splenocin\_biosynthetic\_gene\_cluster | 30 | 43.2586946165 | 331 | 7.6e-90 |
| AEF33079.1 | polyketide\_synthase | BGC0001039 | Pyridomycin\_biosynthetic\_gene\_cluster | 29 | 43.0681276798 | 330 | 1.7e-89 |
| AIW00670.1 | mellein\_synthase | BGC0001244 | (-)-Mellein\_biosynthetic\_gene\_cluster | 28 | 42.9728442115 | 329 | 3.8e-89 |
| AAQ84144.1 | Plm4 | BGC0000123 | Phoslactomycin\_B\_biosynthetic\_gene\_cluster | 28 | 41.4483087184 | 329 | 3.8e-89 |
| CAJ76298.1 | putative\_hybrid\_polyketide-non-ribosomal\_peptide\_synthetase | BGC0000972 | Colibactin\_biosynthetic\_gene\_cluster | 28 | 42.9252024774 | 326 | 2.4e-88 |
| WP\_010639241.1 | beta-ketoacyl\_synthase | BGC0000958 | Antimycin\_biosynthetic\_gene\_cluster | 30 | 43.115769414 | 325 | 5.4e-88 |
| AMY15068.1 | hexaketide\_synthase\_MF-SQHKS | BGC0001339 | Squalestatin\_S1\_biosynthetic\_gene\_cluster | 29 | 39.6855645545 | 324 | 7.1e-88 |
| AFV52200.1 | polyketide\_synthase\_module | BGC0000081 | Kedarcidin\_biosynthetic\_gene\_cluster | 29 | 43.8780371606 | 323 | 1.6e-87 |
| AJO72735.1 | Type\_I\_modular\_polyketide\_synthase | BGC0001381 | Brasilinolide\_biosynthetic\_gene\_cluster | 28 | 47.2129585517 | 322 | 2.7e-87 |
| ADU85988.1 | putative\_iterative\_type\_I\_polyketide\_synthase | BGC0000165 | Tiacumicin\_B\_biosynthetic\_gene\_cluster | 29 | 43.0204859457 | 322 | 3.5e-87 |
| ALJ49910.1 | TlmH | BGC0001237 | Thiolactomycin\_biosynthetic\_gene\_cluster | 28 | 47.594092425 | 321 | 6e-87 |
| AGC09485.1 | LobS2 | BGC0001183 | Lobophorin\_biosynthetic\_gene\_cluster | 28 | 44.9261553121 | 320 | 1.7e-86 |
| AGO86662.1 | equisetin\_synthetase | BGC0001255 | Equisetin\_biosynthetic\_gene\_cluster | 30 | 38.3039542639 | 319 | 2.3e-86 |
| CBF87072.1 | polyketide\_synthase,\_putative\_(Eurofung) | BGC0001290 | Emericellamide\_A\_/\_emericellamide\_B\_biosynthetic\_gene\_cluster | 29 | 45.0690805145 | 319 | 3e-86 |
| CUI25727.1 | Polyketide\_synthase/Nonribosomal\_peptide\_synthase | BGC0001355 | U-68204\_biosynthetic\_gene\_cluster | 28 | 47.8323010958 | 319 | 3e-86 |
| AAG02357.1 | polyketide\_synthase | BGC0000963 | Bleomycin\_biosynthetic\_gene\_cluster | 30 | 42.9252024774 | 319 | 3e-86 |
| AAC68815.1 | FK506\_polyketide\_synthase | BGC0000353 | FK506\_biosynthetic\_gene\_cluster | 29 | 43.0204859457 | 318 | 5.1e-86 |
| ADI59533.1 | CorK | BGC0001091 | Corallopyronin\_biosynthetic\_gene\_cluster | 36 | 25.1071939019 | 318 | 5.1e-86 |
| ADI59531.1 | CorI | BGC0001091 | Corallopyronin\_biosynthetic\_gene\_cluster | 37 | 25.0595521677 | 318 | 6.6e-86 |
| ALJ49921.1 | TtmH | BGC0001236 | Thiotetroamide\_biosynthetic\_gene\_cluster | 27 | 51.9294902334 | 317 | 1.5e-85 |
| ABC36203.1 | thiotemplate\_mechanism\_natural\_product\_synthetase | BGC0001102 | Malleilactone\_biosynthetic\_gene\_cluster | 29 | 45.212005717 | 317 | 1.5e-85 |
| CBJ89766.1 | Polyketide\_synthase\_involved\_in\_xenocoumacin\_synthesis | BGC0001054 | Xenocoumacin\_biosynthetic\_gene\_cluster | 29 | 42.8299190091 | 316 | 2.5e-85 |
| ACZ57548.1 | polyketide\_synthase | BGC0000046 | Depudecin\_biosynthetic\_gene\_cluster | 30 | 41.6388756551 | 312 | 3.6e-84 |
| AIG62146.1 | 6-methylsalicylic\_acid\_synthase | BGC0000120 | Patulin\_biosynthetic\_gene\_cluster | 28 | 41.92472606 | 312 | 3.6e-84 |
| EHA22196.1 | polyketide\_synthase | BGC0000170 | Yanuthone\_D\_biosynthetic\_gene\_cluster | 31 | 35.8265840877 | 310 | 1.4e-83 |
| CUI25744.1 | Polyketide\_synthase/Nonribosomal\_peptide\_synthase | BGC0001354 | U-68204\_biosynthetic\_gene\_cluster | 27 | 51.6912815626 | 308 | 6.9e-83 |
| AAY32964.1 | DszA | BGC0001093 | Disorazole\_biosynthetic\_gene\_cluster | 33 | 27.2034302049 | 308 | 6.9e-83 |
| CCA89326.1 | mixed\_trans-AT\_type\_I\_polyketide\_synthase/nonribosomal\_peptide\_synthetase | BGC0001111 | Rhizopodins\_biosynthetic\_gene\_cluster | 36 | 24.6784182944 | 308 | 6.9e-83 |
| AFR69333.1 | polyketide\_synthase\_SpiC1 | BGC0001045 | Spiruchostatin\_biosynthetic\_gene\_cluster | 37 | 24.4878513578 | 308 | 6.9e-83 |
| AHV78252.1 | ResS1 | BGC0001246 | Trans-resorcylide\_biosynthetic\_gene\_cluster | 29 | 44.1638875655 | 307 | 9e-83 |
| CAO91861.1 | putative\_PKS-NRPS\_protein | BGC0000968 | Chaetoglobosin\_biosynthetic\_gene\_cluster | 28 | 42.6869938066 | 305 | 3.4e-82 |
| ALD83703.1 | tAT\_polyketide\_synthase | BGC0001299 | Chlorotonil\_biosynthetic\_gene\_cluster | 35 | 25.2977608385 | 305 | 4.5e-82 |
| AHB38509.1 | EpnH | BGC0000345 | Eponemycin\_biosynthetic\_gene\_cluster | 30 | 42.7346355407 | 305 | 5.8e-82 |
| ADH04659.1 | TugC | BGC0001342 | Thuggacin\_biosynthetic\_gene\_cluster | 27 | 50.9290138161 | 304 | 9.9e-82 |
| ABC34832.1 | polyketide\_synthase | BGC0000186 | Thailandamide\_biosynthetic\_gene\_cluster | 37 | 24.7737017627 | 304 | 9.9e-82 |
| ADA82581.1 | trans-AT\_polyketide\_synthase | BGC0001110 | Psymberin\_biosynthetic\_gene\_cluster | 36 | 23.058599333 | 304 | 1.3e-81 |
| AIU36103.1 | LglD | BGC0000180 | Legioliulin\_biosynthetic\_gene\_cluster | 33 | 24.6784182944 | 302 | 3.8e-81 |
| CAQ34917.1 | polyketide\_synthase | BGC0000986 | Dkxanthene\_biosynthetic\_gene\_cluster | 33 | 31.443544545 | 302 | 3.8e-81 |
| AMH40443.1 | PKS | BGC0001350 | Phormidolide\_biosynthetic\_gene\_cluster | 34 | 26.0123868509 | 302 | 4.9e-81 |
| ABI91465.1 | beta-ketoacyl\_synthase | BGC0001094 | Enacyloxin\_biosynthetic\_gene\_cluster | 36 | 25.3454025727 | 300 | 1.1e-80 |
| CBJ89760.1 | Polyketide\_synthase\_involved\_in\_xenocoumacin\_synthesis | BGC0001054 | Xenocoumacin\_biosynthetic\_gene\_cluster | 27 | 40.0190566937 | 300 | 1.4e-80 |
| CBF80487.1 | hybrid\_PKS-NRPS\_(Eurofung) | BGC0000959 | Aspyridone\_biosynthetic\_gene\_cluster | 26 | 49.4521200572 | 299 | 3.2e-80 |
| CAN93348.1 | polyketide\_synthase | BGC0000179 | Etnangien\_biosynthetic\_gene\_cluster | 34 | 25.154835636 | 298 | 5.5e-80 |
| ALD83702.1 | tAT\_polyketide\_synthase | BGC0001299 | Chlorotonil\_biosynthetic\_gene\_cluster | 35 | 25.2977608385 | 297 | 9.3e-80 |
| CCA89328.1 | mixed\_trans-AT\_type\_I\_polyketide\_synthase/nonribosomal\_peptide\_synthetase | BGC0001111 | Rhizopodins\_biosynthetic\_gene\_cluster | 33 | 24.8213434969 | 297 | 1.6e-79 |
| CAG23977.1 | polyketide\_synthase\_type\_I | BGC0000176 | Difficidin\_biosynthetic\_gene\_cluster | 33 | 26.1553120534 | 296 | 2.7e-79 |
| AEC04361.1 | polyketide\_synthase | BGC0000178 | Elansolid\_biosynthetic\_gene\_cluster | 35 | 24.3925678895 | 296 | 2.7e-79 |
| AJO72743.1 | Type\_I\_modular\_polyketide\_synthase | BGC0001381 | Brasilinolide\_biosynthetic\_gene\_cluster | 27 | 44.2115292997 | 295 | 3.5e-79 |
| AAP33839.2 | ochratoxin\_A\_polyketide\_synthase | BGC0001030 | Ochratoxin\_A\_biosynthetic\_gene\_cluster | 29 | 35.6836588852 | 295 | 4.6e-79 |
| ADN68483.1 | SorH | BGC0000184 | Sorangicin\_biosynthetic\_gene\_cluster | 36 | 25.5359695093 | 294 | 7.9e-79 |
| BAP05594.1 | CalF | BGC0000967 | Calyculin\_biosynthetic\_gene\_cluster | 33 | 25.7741781801 | 294 | 1e-78 |
| AIJ04680.1 | polyketide\_synthase | BGC0001383 | Macrolactin\_biosynthetic\_gene\_cluster | 34 | 25.154835636 | 293 | 1.8e-78 |
| AFN27482.1 | pks\_BonC | BGC0000173 | Bongkrekic\_acid\_biosynthetic\_gene\_cluster | 34 | 25.2024773702 | 293 | 1.8e-78 |
| AMH40422.1 | PKS | BGC0001350 | Phormidolide\_biosynthetic\_gene\_cluster | 33 | 26.2029537875 | 293 | 2.3e-78 |
| BAF50727.1 | hybrid\_polyketide\_synthase-non\_ribosomal\_peptide\_synthetase | BGC0001116 | Virginiamycin\_biosynthetic\_gene\_cluster | 34 | 25.5836112434 | 293 | 2.3e-78 |
| AIC32694.1 | FR9GH | BGC0001113 | Spliceostatin\_/\_FR901464\_biosynthetic\_gene\_cluster | 33 | 24.8213434969 | 292 | 3.9e-78 |
| ADH01489.1 | type\_I\_polyketide\_synthase | BGC0001096 | FR901464\_biosynthetic\_gene\_cluster | 33 | 24.8213434969 | 292 | 3.9e-78 |
| AAY89053.1 | polyketide\_synthase | BGC0001069 | Chivosazole\_biosynthetic\_gene\_cluster | 36 | 23.2491662697 | 292 | 3.9e-78 |
| AGN11882.1 | TstGH | BGC0001114 | Thailanstatin\_biosynthetic\_gene\_cluster | 33 | 25.3454025727 | 291 | 6.7e-78 |
| CAJ57411.1 | polyketide\_synthase\_type\_I | BGC0000176 | Difficidin\_biosynthetic\_gene\_cluster | 33 | 26.9175797999 | 291 | 6.7e-78 |
| ABF85931.1 | non-ribosomal\_peptide\_synthase/polyketide\_synthase\_Ta1 | BGC0001025 | Myxovirescin\_biosynthetic\_gene\_cluster | 35 | 24.9642686994 | 291 | 6.7e-78 |
| CAG23965.1 | polyketide\_synthase\_type\_I | BGC0000181 | Macrolactin\_biosynthetic\_gene\_cluster | 34 | 24.7737017627 | 291 | 8.7e-78 |
| CTQ34881.1 | AtcD;\_polyketide\_synthase,\_modules\_1-4 | BGC0001301 | Anthracimycin\_biosynthetic\_gene\_cluster | 34 | 26.34587899 | 291 | 8.7e-78 |
| AKQ22698.1 | malonyl\_CoA-acyl\_carrier\_protein\_transacylase | BGC0001186 | Misakinolide\_biosynthetic\_gene\_cluster | 34 | 25.2024773702 | 290 | 1.1e-77 |
| CAN93347.1 | Polyketide\_synthase | BGC0000179 | Etnangien\_biosynthetic\_gene\_cluster | 34 | 26.060028585 | 290 | 1.1e-77 |
| CAG23958.2 | polyketide\_synthase | BGC0001089 | Bacillaene\_biosynthetic\_gene\_cluster | 35 | 24.8213434969 | 290 | 1.1e-77 |
| BAP05595.1 | CalG | BGC0000967 | Calyculin\_biosynthetic\_gene\_cluster | 34 | 25.4883277751 | 290 | 1.1e-77 |
| ADN68476.1 | SorA | BGC0000184 | Sorangicin\_biosynthetic\_gene\_cluster | 34 | 24.6307765603 | 290 | 1.5e-77 |
| ADN68477.1 | SorB | BGC0000184 | Sorangicin\_biosynthetic\_gene\_cluster | 33 | 25.9647451167 | 290 | 1.5e-77 |
| AFN27483.1 | pks\_BonD | BGC0000173 | Bongkrekic\_acid\_biosynthetic\_gene\_cluster | 34 | 25.0119104335 | 290 | 1.9e-77 |
| ADA82585.1 | hybrid\_trans-AT\_polyketide\_synthase\_-\_nonribosomal\_peptide\_synthetase | BGC0001110 | Psymberin\_biosynthetic\_gene\_cluster | 35 | 26.6793711291 | 289 | 2.5e-77 |
| ACY01391.1 | AT-less\_polyketide\_synthase | BGC0000177 | Dorrigocin\_/\_migrastatin\_biosynthetic\_gene\_cluster | 36 | 25.0119104335 | 289 | 2.5e-77 |
| ALD82523.1 | polyketide\_synthase | BGC0001212 | Nannocystin\_biosynthetic\_gene\_cluster | 40 | 21.3434969033 | 289 | 3.3e-77 |
| CTQ34882.1 | AtcE;\_polyketide\_synthase,\_modules\_5-7 | BGC0001301 | Anthracimycin\_biosynthetic\_gene\_cluster | 36 | 21.8199142449 | 288 | 5.6e-77 |
| EAU29808.1 | hypothetical\_protein | BGC0001400 | Citreoviridin\_biosynthetic\_gene\_cluster | 29 | 44.8308718437 | 288 | 5.6e-77 |
| ABF92489.1 | mixed\_type\_I\_polyketide\_synthase\_-\_peptide\_synthetase | BGC0001025 | Myxovirescin\_biosynthetic\_gene\_cluster | 33 | 26.6793711291 | 288 | 5.6e-77 |
| CCT72377.1 | probable\_polyketide\_synthase | BGC0001305 | Fujikurins\_biosynthetic\_gene\_cluster | 26 | 55.4073368271 | 287 | 9.6e-77 |
| AAM12911.1 | MmpB | BGC0000182 | Mupirocin\_biosynthetic\_gene\_cluster | 34 | 25.2501191043 | 287 | 9.6e-77 |
| ABC33986.1 | polyketide\_synthase,\_putative | BGC0000186 | Thailandamide\_biosynthetic\_gene\_cluster | 32 | 28.0609814197 | 287 | 1.3e-76 |
| ALD83686.1 | tAT\_polyketide\_synthase | BGC0001300 | Anthracimycin\_biosynthetic\_gene\_cluster | 35 | 24.5354930919 | 287 | 1.3e-76 |
| CAE14178.1 |  | BGC0001180 | Isopropylstilbene\_biosynthetic\_gene\_cluster | 35 | 24.3449261553 | 287 | 1.6e-76 |
| ABS90470.1 | NRPS/PKS | BGC0001106 | Oxazolomycin\_biosynthetic\_gene\_cluster | 37 | 23.6779418771 | 286 | 2.1e-76 |
| AFB35635.1 | EsmB4 | BGC0000935 | Esmeraldin\_biosynthetic\_gene\_cluster | 31 | 33.3015721772 | 286 | 2.8e-76 |
| AFN27480.1 | pks\_BonA | BGC0000173 | Bongkrekic\_acid\_biosynthetic\_gene\_cluster | 33 | 25.2977608385 | 285 | 3.7e-76 |
| CBK62724.1 |  | BGC0001115 | Thiomarinol\_biosynthetic\_gene\_cluster | 33 | 24.1067174845 | 285 | 3.7e-76 |
| AFX60313.1 | polyketide\_synthase | BGC0001031 | Oocydin\_A\_biosynthetic\_gene\_cluster | 34 | 25.6312529776 | 285 | 3.7e-76 |
| AKQ22696.1 | malonyl\_CoA-acyl\_carrier\_protein\_transacylase | BGC0001186 | Misakinolide\_biosynthetic\_gene\_cluster | 35 | 25.440686041 | 285 | 4.8e-76 |
| ABS90471.1 | PKS\_type\_I | BGC0001106 | Oxazolomycin\_biosynthetic\_gene\_cluster | 33 | 24.8689852311 | 285 | 4.8e-76 |
| ADI59534.1 | CorL | BGC0001091 | Corallopyronin\_biosynthetic\_gene\_cluster | 34 | 24.9642686994 | 285 | 4.8e-76 |
| CAN93350.1 | polyketide\_synthase | BGC0000179 | Etnangien\_biosynthetic\_gene\_cluster | 38 | 20.8670795617 | 285 | 4.8e-76 |
| CAG23983.1 | polyketide\_synthase\_type\_I | BGC0000176 | Difficidin\_biosynthetic\_gene\_cluster | 35 | 25.4883277751 | 284 | 8.1e-76 |
| CAG23959.2 | polyketide\_synthase | BGC0001089 | Bacillaene\_biosynthetic\_gene\_cluster | 33 | 26.1076703192 | 284 | 8.1e-76 |
| BAP05593.1 | CalE | BGC0000967 | Calyculin\_biosynthetic\_gene\_cluster | 34 | 25.1071939019 | 284 | 8.1e-76 |
| ABM63528.1 | BryC | BGC0000174 | Bryostatin\_biosynthetic\_gene\_cluster | 35 | 25.3930443068 | 284 | 1.1e-75 |
| CAG23978.1 | polyketide\_synthase\_type\_I | BGC0000176 | Difficidin\_biosynthetic\_gene\_cluster | 33 | 25.4883277751 | 284 | 1.1e-75 |
| ABM63527.1 | BryB | BGC0000174 | Bryostatin\_biosynthetic\_gene\_cluster | 35 | 25.3930443068 | 284 | 1.1e-75 |
| CAG23967.1 | polyketide\_synthase\_type\_I | BGC0000181 | Macrolactin\_biosynthetic\_gene\_cluster | 33 | 24.8689852311 | 284 | 1.4e-75 |
| AIJ04684.1 | polyketide\_synthase | BGC0001383 | Macrolactin\_biosynthetic\_gene\_cluster | 33 | 25.154835636 | 283 | 1.8e-75 |
| AAN85523.1 | polyketide\_synthase | BGC0001101 | Leinamycin\_biosynthetic\_gene\_cluster | 35 | 24.7260600286 | 283 | 2.4e-75 |
| ADD82941.1 | Bat3 | BGC0001099 | Kalimantacin\_/\_batumin\_biosynthetic\_gene\_cluster | 33 | 25.440686041 | 283 | 2.4e-75 |
| AFN27481.1 | pks\_BonB | BGC0000173 | Bongkrekic\_acid\_biosynthetic\_gene\_cluster | 33 | 25.5359695093 | 282 | 3.1e-75 |
| AGN11881.1 | TstDEF | BGC0001114 | Thailanstatin\_biosynthetic\_gene\_cluster | 31 | 28.8708909004 | 282 | 4e-75 |
| ALD83688.1 | tAT\_polyketide\_synthase | BGC0001300 | Anthracimycin\_biosynthetic\_gene\_cluster | 32 | 25.3930443068 | 282 | 5.3e-75 |
| AAM12909.2 | MmpA | BGC0000182 | Mupirocin\_biosynthetic\_gene\_cluster | 35 | 25.0119104335 | 282 | 5.3e-75 |
| ABC35522.1 | thiotemplate\_mechanism\_natural\_product\_synthetase | BGC0000186 | Thailandamide\_biosynthetic\_gene\_cluster | 34 | 25.6788947118 | 282 | 5.3e-75 |
| ABC34675.1 | polyketide\_synthase,\_putative | BGC0000186 | Thailandamide\_biosynthetic\_gene\_cluster | 33 | 25.3930443068 | 282 | 5.3e-75 |
| CAL69894.1 | RhiF\_protein | BGC0001112 | Rhizoxins\_biosynthetic\_gene\_cluster | 33 | 23.9161505479 | 281 | 6.9e-75 |
| CAG23957.2 | NRPS/PKS\_protein | BGC0001089 | Bacillaene\_biosynthetic\_gene\_cluster | 31 | 26.4411624583 | 281 | 6.9e-75 |
| AAS47562.1 | mixed\_type\_I\_polyketide\_synthase\_-\_peptide\_synthetase | BGC0001108 | Pederin\_biosynthetic\_gene\_cluster | 33 | 25.3930443068 | 281 | 9e-75 |
| CAG23964.1 | polyketide\_synthase\_type\_I | BGC0000181 | Macrolactin\_biosynthetic\_gene\_cluster | 32 | 27.2034302049 | 281 | 9e-75 |
| CBF69451.1 | polyketide\_synthase,\_putative\_(JCVI) | BGC0000037 | Cichorine\_biosynthetic\_gene\_cluster | 34 | 29.9190090519 | 280 | 1.2e-74 |
| AGS06824.1 | mixed\_type\_I\_polyketide\_synthase/non-ribosomal\_peptide\_synthetase,\_PedH-like\_protein | BGC0001092 | Diaphorin\_biosynthetic\_gene\_cluster | 33 | 24.3925678895 | 280 | 1.5e-74 |
| CAL69890.1 | RhiC\_protein | BGC0001112 | Rhizoxins\_biosynthetic\_gene\_cluster | 34 | 24.1543592187 | 280 | 2e-74 |
| AFX60334.1 | polyketide\_synthase | BGC0001032 | Oocydin\_A\_biosynthetic\_gene\_cluster | 33 | 24.6784182944 | 279 | 2.6e-74 |
| CBK62733.1 |  | BGC0001115 | Thiomarinol\_biosynthetic\_gene\_cluster | 34 | 24.2972844212 | 279 | 3.4e-74 |
| AFX60311.1 | polyketide\_synthase | BGC0001031 | Oocydin\_A\_biosynthetic\_gene\_cluster | 34 | 25.3454025727 | 279 | 3.4e-74 |
| CBW45749.1 | hybrid\_PKS/NRPS\_(PKSI) | BGC0000952\_c3 | Pristinamycin\_biosynthetic\_gene\_cluster | 34 | 25.9647451167 | 279 | 3.4e-74 |
| AFX60317.1 | polyketide\_synthase | BGC0001031 | Oocydin\_A\_biosynthetic\_gene\_cluster | 33 | 25.9647451167 | 279 | 3.4e-74 |
| AKA59437.1 | polyketide\_synthase | BGC0001202 | Landepoxcin\_biosynthetic\_gene\_cluster | 29 | 38.4945212006 | 279 | 3.4e-74 |
| ALD83704.1 | tAT\_polyketide\_synthase | BGC0001299 | Chlorotonil\_biosynthetic\_gene\_cluster | 35 | 22.9633158647 | 279 | 4.5e-74 |
| AKQ22699.1 | malonyl\_CoA-acyl\_carrier\_protein\_transacylase | BGC0001186 | Misakinolide\_biosynthetic\_gene\_cluster | 33 | 25.154835636 | 278 | 5.8e-74 |
| AIJ04681.1 | polyketide\_synthase | BGC0001383 | Macrolactin\_biosynthetic\_gene\_cluster | 32 | 27.2034302049 | 278 | 5.8e-74 |
| AEH42473.1 | polyketide\_synthase | BGC0000032 | Calcimycin\_biosynthetic\_gene\_cluster | 32 | 34.8261076703 | 278 | 5.8e-74 |
| AFX60336.1 | polyketide\_synthase | BGC0001032 | Oocydin\_A\_biosynthetic\_gene\_cluster | 33 | 25.6788947118 | 278 | 5.8e-74 |
| ADD82940.1 | Bat2 | BGC0001099 | Kalimantacin\_/\_batumin\_biosynthetic\_gene\_cluster | 32 | 27.251071939 | 278 | 7.6e-74 |
| ADA69237.1 | trans-AT\_polyketide\_synthase | BGC0001071 | Nosperin\_biosynthetic\_gene\_cluster | 32 | 27.536922344 | 277 | 1e-73 |
| ACY01401.1 | AT-less\_polyketide\_synthase | BGC0000083 | Lactimidomycin\_biosynthetic\_gene\_cluster | 32 | 30.3954263935 | 277 | 1e-73 |
| ADN68480.1 | SorE | BGC0000184 | Sorangicin\_biosynthetic\_gene\_cluster | 34 | 25.2977608385 | 277 | 1.7e-73 |
| CAG23960.2 | hybrid\_NRPS/PKS | BGC0001089 | Bacillaene\_biosynthetic\_gene\_cluster | 33 | 24.8213434969 | 276 | 2.2e-73 |
| AHE80993.1 | PieA3 | BGC0001169 | Piericidin\_A1\_biosynthetic\_gene\_cluster | 30 | 31.7770366841 | 276 | 2.9e-73 |
| ACY01392.1 | AT-less\_polyketide\_synthase | BGC0000177 | Dorrigocin\_/\_migrastatin\_biosynthetic\_gene\_cluster | 33 | 25.0119104335 | 275 | 3.8e-73 |
| AAY89050.1 | polyketide\_synthase | BGC0001069 | Chivosazole\_biosynthetic\_gene\_cluster | 35 | 25.6788947118 | 275 | 3.8e-73 |
| CCA89327.1 | trans-AT\_type\_I\_polyketide\_synthase | BGC0001111 | Rhizopodins\_biosynthetic\_gene\_cluster | 33 | 25.154835636 | 275 | 4.9e-73 |
| CAG23968.1 | polyketide\_synthase\_type\_I | BGC0000181 | Macrolactin\_biosynthetic\_gene\_cluster | 31 | 25.4883277751 | 275 | 6.5e-73 |
| ALD83687.1 | tAT\_polyketide\_synthase | BGC0001300 | Anthracimycin\_biosynthetic\_gene\_cluster | 32 | 24.9642686994 | 275 | 6.5e-73 |
| ERM18797.1 | polyketide\_synthase | BGC0000172 | Basiliskamides\_biosynthetic\_gene\_cluster | 31 | 26.7270128633 | 275 | 6.5e-73 |
| AEC04357.1 | polyketide\_synthase | BGC0000178 | Elansolid\_biosynthetic\_gene\_cluster | 33 | 25.2501191043 | 274 | 8.4e-73 |
| AIJ04686.1 | polyketide\_synthase | BGC0001383 | Macrolactin\_biosynthetic\_gene\_cluster | 31 | 25.3930443068 | 274 | 1.1e-72 |
| AIC32693.1 | FR9DEF | BGC0001113 | Spliceostatin\_/\_FR901464\_biosynthetic\_gene\_cluster | 34 | 24.7260600286 | 274 | 1.4e-72 |
| ADH01487.1 | polyketide\_synthase | BGC0001096 | FR901464\_biosynthetic\_gene\_cluster | 34 | 24.7260600286 | 274 | 1.4e-72 |
| ACR13065.1 | modular\_polyketide\_synthase,\_type\_I\_PKS | BGC0000185 | Tartrolon\_biosynthetic\_gene\_cluster | 31 | 26.5364459266 | 273 | 1.9e-72 |
| AGS06887.1 | mixed\_type\_I\_polyketide\_synthase/non-ribosomal\_peptide\_synthetase,\_PedF-like\_protein | BGC0001092\_c2 | Diaphorin\_biosynthetic\_gene\_cluster | 33 | 24.5354930919 | 273 | 1.9e-72 |
| ABC34176.1 | polyketide\_synthase,\_putative | BGC0000186 | Thailandamide\_biosynthetic\_gene\_cluster | 36 | 20.4859456884 | 273 | 1.9e-72 |
| CCC21123.1 | type-I\_polyketide\_synthases | BGC0000171 | 9-methylstreptimidone\_biosynthetic\_gene\_cluster | 32 | 25.3454025727 | 272 | 3.2e-72 |
| ADI59532.1 | CorJ | BGC0001091 | Corallopyronin\_biosynthetic\_gene\_cluster | 34 | 24.3449261553 | 272 | 3.2e-72 |
| AGS06823.1 | type\_I\_polyketide\_synthase,\_PedI-like\_protein | BGC0001092 | Diaphorin\_biosynthetic\_gene\_cluster | 32 | 24.6784182944 | 272 | 5.5e-72 |
| ABF92824.1 | polyketide\_synthase | BGC0001025 | Myxovirescin\_biosynthetic\_gene\_cluster | 32 | 24.8213434969 | 272 | 5.5e-72 |
| CTQ34883.1 | AtcF;\_polyketide\_synthase,\_modules\_8-10 | BGC0001301 | Anthracimycin\_biosynthetic\_gene\_cluster | 32 | 25.2977608385 | 272 | 5.5e-72 |
| ctg1\_orf6 |  | BGC0001109 | Pederin\_biosynthetic\_gene\_cluster | 32 | 24.7260600286 | 272 | 5.5e-72 |
| BAD83684.1 | PKSN\_polyketide\_synthase\_for\_alternapyrone\_biosynthesis | BGC0000012 | Alternapyrone\_biosynthetic\_gene\_cluster | 36 | 20.6765126251 | 272 | 5.5e-72 |
| AAS47564.1 | mixed\_type\_I\_polyketide\_synthase/nonribosomal\_peptide\_synthetase | BGC0001108 | Pederin\_biosynthetic\_gene\_cluster | 32 | 24.7260600286 | 272 | 5.5e-72 |
| CAJ57409.1 | polyketide\_synthase\_type\_I | BGC0000176 | Difficidin\_biosynthetic\_gene\_cluster | 34 | 26.4888041925 | 271 | 7.1e-72 |
| ABI91469.1 | beta-ketoacyl\_synthase | BGC0001094 | Enacyloxin\_biosynthetic\_gene\_cluster | 37 | 22.6298237256 | 271 | 7.1e-72 |
| AMH40421.1 | PKS | BGC0001350 | Phormidolide\_biosynthetic\_gene\_cluster | 33 | 26.1076703192 | 271 | 9.3e-72 |
| AEC04356.1 | polyketide\_synthase | BGC0000178 | Elansolid\_biosynthetic\_gene\_cluster | 32 | 24.5831348261 | 271 | 9.3e-72 |
| CAN89631.1 | putative\_polyketide\_synthase | BGC0001070 | Kirromycin\_biosynthetic\_gene\_cluster | 34 | 25.440686041 | 271 | 9.3e-72 |
| CCA89329.1 | trans-AT\_type\_I\_polyketide\_synthase | BGC0001111 | Rhizopodins\_biosynthetic\_gene\_cluster | 31 | 29.2520247737 | 271 | 9.3e-72 |
| AFX60340.1 | polyketide\_synthase | BGC0001032 | Oocydin\_A\_biosynthetic\_gene\_cluster | 32 | 25.2501191043 | 270 | 1.2e-71 |
| CAN89634.1 | putative\_polyketide\_synthase | BGC0001070 | Kirromycin\_biosynthetic\_gene\_cluster | 38 | 21.6769890424 | 270 | 1.6e-71 |
| CAN89632.1 | putative\_polyketide\_synthase | BGC0001070 | Kirromycin\_biosynthetic\_gene\_cluster | 33 | 25.4883277751 | 270 | 2.1e-71 |
| AFX60332.1 | polyketide\_synthase | BGC0001032 | Oocydin\_A\_biosynthetic\_gene\_cluster | 32 | 25.6312529776 | 269 | 2.7e-71 |
| AMH40423.1 | PKS | BGC0001350 | Phormidolide\_biosynthetic\_gene\_cluster | 33 | 25.4883277751 | 269 | 4.6e-71 |
| ACY01402.1 | AT-less\_polyketide\_synthase | BGC0000083 | Lactimidomycin\_biosynthetic\_gene\_cluster | 32 | 26.7746545974 | 269 | 4.6e-71 |
| AAY32965.1 | DszB | BGC0001093 | Disorazole\_biosynthetic\_gene\_cluster | 34 | 25.1071939019 | 268 | 6e-71 |
| CAN93351.1 | polyketide\_synthase | BGC0000179 | Etnangien\_biosynthetic\_gene\_cluster | 32 | 25.0119104335 | 268 | 6e-71 |
| AGN74893.1 | nonribosomal\_peptide\_synthetase/polyketide\_synthase\_hybrid\_protein | BGC0000459 | Griseoviridin\_/\_viridogrisein\_biosynthetic\_gene\_cluster | 32 | 25.7741781801 | 267 | 1e-70 |
| AMY15057.1 | tetraketide\_synthase\_MF-SQTKS | BGC0001339 | Squalestatin\_S1\_biosynthetic\_gene\_cluster | 35 | 20.6765126251 | 267 | 1.3e-70 |
| ACR12418.1 | modular\_polyketide\_synthase,\_type\_I\_PKS | BGC0000185 | Tartrolon\_biosynthetic\_gene\_cluster | 30 | 29.9190090519 | 266 | 2.3e-70 |
| CAL69889.1 | RhiB\_protein | BGC0001112 | Rhizoxins\_biosynthetic\_gene\_cluster | 33 | 25.3454025727 | 266 | 2.3e-70 |
| AFX60309.1 | polyketide\_synthase | BGC0001031 | Oocydin\_A\_biosynthetic\_gene\_cluster | 32 | 25.8218199142 | 266 | 2.3e-70 |
| CBW45748.1 | hybrid\_PKS/NRPS\_(PKSI) | BGC0000952\_c3 | Pristinamycin\_biosynthetic\_gene\_cluster | 32 | 25.2501191043 | 265 | 5.1e-70 |
| CAE52339.1 | Polyketide\_non-ribosomal\_peptide\_synthase | BGC0001088 | Albicidin\_biosynthetic\_gene\_cluster | 31 | 25.3930443068 | 265 | 5.1e-70 |
| AAM12913.2 | MmpD | BGC0000182 | Mupirocin\_biosynthetic\_gene\_cluster | 31 | 24.9642686994 | 265 | 6.7e-70 |
| ABM63529.1 | BryX | BGC0000174 | Bryostatin\_biosynthetic\_gene\_cluster | 33 | 25.0119104335 | 265 | 6.7e-70 |
| AEH59109.1 | keto-hydroxyglutarate-aldolase/polyketide\_synthase | BGC0000385 | Lysobactin\_biosynthetic\_gene\_cluster | 33 | 25.3930443068 | 264 | 1.1e-69 |
| CAN89635.1 | putative\_polyketide\_synthase | BGC0001070 | Kirromycin\_biosynthetic\_gene\_cluster | 33 | 22.4868985231 | 264 | 1.5e-69 |
| CAN93349.1 | polyketide\_synthase | BGC0000179 | Etnangien\_biosynthetic\_gene\_cluster | 31 | 29.0614578371 | 263 | 1.9e-69 |
| AGN74892.1 | nonribosomal\_peptide\_synthetase/polyketide\_synthase\_hybrid\_protein | BGC0000459 | Griseoviridin\_/\_viridogrisein\_biosynthetic\_gene\_cluster | 34 | 25.6312529776 | 263 | 2.5e-69 |
| ANF07288.1 | hrPKS | BGC0001340 | Byssochlamic\_acid\_biosynthetic\_gene\_cluster | 36 | 21.0576464983 | 263 | 2.5e-69 |
| CAN93352.1 | polyketide\_synthase | BGC0000179 | Etnangien\_biosynthetic\_gene\_cluster | 33 | 25.8694616484 | 263 | 2.5e-69 |
| AHE80996.1 | PieA6 | BGC0001169 | Piericidin\_A1\_biosynthetic\_gene\_cluster | 35 | 21.0576464983 | 262 | 3.3e-69 |
| ALD82521.1 | polyketide\_synthase | BGC0001212 | Nannocystin\_biosynthetic\_gene\_cluster | 35 | 21.8199142449 | 262 | 5.7e-69 |
| CAJ57408.1 | polyketide\_synthase\_type\_I | BGC0000176 | Difficidin\_biosynthetic\_gene\_cluster | 32 | 23.4873749404 | 261 | 7.4e-69 |
| BAC76474.1 | type\_I\_polyketide\_synthase\_LkcC | BGC0001100 | Lankacidin\_biosynthetic\_gene\_cluster | 33 | 24.9166269652 | 261 | 7.4e-69 |
| EWG54266.1 | hypothetical\_protein | BGC0001190 | Fusaric\_acid\_biosynthetic\_gene\_cluster | 35 | 20.8670795617 | 261 | 9.6e-69 |
| AEC04363.1 | polyketide\_synthase | BGC0000178 | Elansolid\_biosynthetic\_gene\_cluster | 31 | 28.1086231539 | 261 | 9.6e-69 |
| CAN89633.1 | putative\_hybrid\_non-ribosomal\_peptide\_synthetase/polyketide\_synthase | BGC0001070 | Kirromycin\_biosynthetic\_gene\_cluster | 37 | 20.9147212959 | 260 | 1.3e-68 |
| CAL69891.1 | RhiD\_protein | BGC0001112 | Rhizoxins\_biosynthetic\_gene\_cluster | 31 | 28.156264888 | 260 | 1.3e-68 |
| ABC87511.1 | polyketide\_synthase | BGC0001011 | Meridamycin\_biosynthetic\_gene\_cluster | 35 | 21.0576464983 | 260 | 1.6e-68 |
| ctg1\_orf22 |  | BGC0001013 | Meridamycin\_biosynthetic\_gene\_cluster | 35 | 21.0576464983 | 260 | 1.6e-68 |
| ACR13997.1 | modular\_polyketide\_synthase,\_type\_I\_PKS | BGC0000185 | Tartrolon\_biosynthetic\_gene\_cluster | 34 | 24.4878513578 | 259 | 2.8e-68 |
| BAP05591.1 | CalC | BGC0000967 | Calyculin\_biosynthetic\_gene\_cluster | 31 | 23.2491662697 | 259 | 2.8e-68 |
| ERM18799.1 | polyketide\_synthase | BGC0000172 | Basiliskamides\_biosynthetic\_gene\_cluster | 31 | 24.9642686994 | 259 | 2.8e-68 |
| ACY01390.1 | AT-less\_polyketide\_synthase | BGC0000177 | Dorrigocin\_/\_migrastatin\_biosynthetic\_gene\_cluster | 33 | 22.8680323964 | 259 | 4.8e-68 |
| BAC57030.1 | protomycinolide\_IV\_synthase\_3 | BGC0000102 | Mycinamicin\_biosynthetic\_gene\_cluster | 35 | 21.0576464983 | 259 | 4.8e-68 |
| AEP40938.1 | polyketide\_synthase\_type\_I | BGC0000021 | Apoptolidin\_biosynthetic\_gene\_cluster | 35 | 20.5812291567 | 259 | 4.8e-68 |
| BAD38875.1 | polyketide\_synthase | BGC0000111 | Neocarzilin\_biosynthetic\_gene\_cluster | 33 | 25.6312529776 | 259 | 4.8e-68 |
| BAC76471.1 | type\_I\_polyketide\_synthase\_LkcF | BGC0001100 | Lankacidin\_biosynthetic\_gene\_cluster | 33 | 24.6784182944 | 258 | 8.2e-68 |
| ADN68478.1 | SorC | BGC0000184 | Sorangicin\_biosynthetic\_gene\_cluster | 33 | 24.9166269652 | 258 | 8.2e-68 |
| CAL69893.1 | RhiE\_protein | BGC0001112 | Rhizoxins\_biosynthetic\_gene\_cluster | 30 | 29.8237255836 | 258 | 8.2e-68 |
| AAL01060.1 | omega-3\_polyunsaturated\_fatty\_acid\_synthase\_PfaA | BGC0000865 | Eicoseicosapentaenoic\_acid\_biosynthetic\_gene\_cluster | 26 | 44.5450214388 | 258 | 8.2e-68 |
| ADH01488.1 | polyketide\_synthase | BGC0001096 | FR901464\_biosynthetic\_gene\_cluster | 31 | 25.0595521677 | 257 | 1.4e-67 |
| BAP05589.1 | CalA | BGC0000967 | Calyculin\_biosynthetic\_gene\_cluster | 33 | 25.4883277751 | 257 | 1.4e-67 |
| BAP05590.1 | CalB | BGC0000967 | Calyculin\_biosynthetic\_gene\_cluster | 33 | 23.8208670796 | 255 | 4.1e-67 |
| AIU36104.1 | LglE | BGC0000180 | Legioliulin\_biosynthetic\_gene\_cluster | 30 | 24.5354930919 | 253 | 1.5e-66 |
| ABK32260.1 | AmbF | BGC0000014 | Ambruticin\_biosynthetic\_gene\_cluster | 33 | 23.4397332063 | 253 | 1.5e-66 |
| AGN74894.1 | nonribosomal\_peptide\_synthetase/polyketide\_synthase\_hybrid\_protein | BGC0000459 | Griseoviridin\_/\_viridogrisein\_biosynthetic\_gene\_cluster | 32 | 25.154835636 | 253 | 2.6e-66 |
| AAO65790.1 | ketosynthase-like\_protein | BGC0000100 | Monensin\_biosynthetic\_gene\_cluster | 28 | 33.3492139114 | 250 | 2.2e-65 |
| BAJ09789.1 | polyketide\_synthase | BGC0000146 | Solanapyrone\_biosynthetic\_gene\_cluster | 36 | 21.5340638399 | 249 | 3.8e-65 |
| AFK83820.1 | PtzD | BGC0001107\_c2 | Patellazole\_biosynthetic\_gene\_cluster | 34 | 23.2968080038 | 248 | 5e-65 |
| AFX60318.1 | polyketide\_synthase | BGC0001031 | Oocydin\_A\_biosynthetic\_gene\_cluster | 32 | 24.6784182944 | 248 | 5e-65 |
| XP\_011392698.1 | hypothetical\_protein | BGC0001281 | Ustilagic\_acid\_biosynthetic\_gene\_cluster | 32 | 26.8699380657 | 248 | 5e-65 |
| AFR69335.1 | polyketide\_synthase\_SpiC2 | BGC0001045 | Spiruchostatin\_biosynthetic\_gene\_cluster | 33 | 24.4878513578 | 248 | 6.5e-65 |
| AIJ04685.1 | polyketide\_synthase | BGC0001383 | Macrolactin\_biosynthetic\_gene\_cluster | 32 | 22.8203906622 | 248 | 6.5e-65 |
| ABF89568.1 | polyketide\_synthase | BGC0001025 | Myxovirescin\_biosynthetic\_gene\_cluster | 31 | 27.3939971415 | 247 | 1.1e-64 |
| CAG23969.1 | polyketide\_synthase\_type\_I | BGC0000181 | Macrolactin\_biosynthetic\_gene\_cluster | 32 | 22.8203906622 | 247 | 1.4e-64 |
| AKQ22697.1 | malonyl\_CoA-acyl\_carrier\_protein\_transacylase | BGC0001186 | Misakinolide\_biosynthetic\_gene\_cluster | 32 | 24.4402096236 | 246 | 3.2e-64 |
| BAF50721.1 | hybrid\_non\_ribosomal\_peptide\_synthetase-polyketide\_synthase | BGC0001116 | Virginiamycin\_biosynthetic\_gene\_cluster | 32 | 25.9171033826 | 246 | 3.2e-64 |
| ABM63537.1 | BryA | BGC0000174 | Bryostatin\_biosynthetic\_gene\_cluster | 31 | 26.2029537875 | 246 | 3.2e-64 |
| ABC35796.1 | polyketide\_synthase,\_putative | BGC0001102 | Malleilactone\_biosynthetic\_gene\_cluster | 36 | 21.0576464983 | 245 | 4.2e-64 |
| CBK62731.1 |  | BGC0001115 | Thiomarinol\_biosynthetic\_gene\_cluster | 30 | 24.9166269652 | 245 | 4.2e-64 |
| ABS90475.1 | PKS | BGC0001106 | Oxazolomycin\_biosynthetic\_gene\_cluster | 31 | 27.4416388757 | 245 | 5.5e-64 |
| ALV82446.1 | putative\_type\_I\_polyketide\_synthase | BGC0001382 | Svaricin\_biosynthetic\_gene\_cluster | 31 | 28.8232491663 | 245 | 5.5e-64 |
| BAK64638.1 | polyketide\_synthase | BGC0000135 | Reveromycin\_biosynthetic\_gene\_cluster | 34 | 22.3439733206 | 245 | 5.5e-64 |
| EPE34340.1 | Thiolase-like\_protein | BGC0001035 | Pneumocandin\_biosynthetic\_gene\_cluster | 31 | 21.4387803716 | 245 | 7.2e-64 |
| AGN11883.1 | TstI | BGC0001114 | Thailanstatin\_biosynthetic\_gene\_cluster | 32 | 25.6312529776 | 243 | 1.6e-63 |
| ADN68479.1 | SorD | BGC0000184 | Sorangicin\_biosynthetic\_gene\_cluster | 30 | 25.3454025727 | 243 | 1.6e-63 |
| AGJ76601.1 | HglE | BGC0000869 | Heterocyst\_glycolipids\_biosynthetic\_gene\_cluster | 28 | 39.3044306813 | 243 | 2.7e-63 |
| EAL85113.2 | hybrid\_PKS-NRPS\_enzyme,\_putative | BGC0001037 | Pseurotin\_A\_biosynthetic\_gene\_cluster | 34 | 20.9147212959 | 242 | 3.5e-63 |
| ACR50796.1 | putative\_polyketide\_synthase | BGC0000163 | Tetronasin\_biosynthetic\_gene\_cluster | 32 | 25.2501191043 | 242 | 4.6e-63 |
| BAP05597.1 | CalI | BGC0000967 | Calyculin\_biosynthetic\_gene\_cluster | 31 | 25.6788947118 | 242 | 4.6e-63 |
| AFL48521.1 | beta-ketosynthase | BGC0000084 | Laidlomycin\_biosynthetic\_gene\_cluster | 29 | 31.2053358742 | 242 | 6.1e-63 |
| ACM79805.1 | ZmaA | BGC0001059 | Zwittermycin\_A\_biosynthetic\_gene\_cluster | 30 | 27.3463554073 | 240 | 1.8e-62 |
| BAP05596.1 | CalH | BGC0000967 | Calyculin\_biosynthetic\_gene\_cluster | 29 | 28.1086231539 | 239 | 3.9e-62 |
| AEC04362.1 | polyketide\_synthase | BGC0000178 | Elansolid\_biosynthetic\_gene\_cluster | 32 | 26.6793711291 | 238 | 6.7e-62 |
| BAE93740.1 | type\_I\_polyketide\_synthase-related\_protein | BGC0000164 | Tetronomycin\_biosynthetic\_gene\_cluster | 33 | 26.0123868509 | 238 | 6.7e-62 |
| CBW45741.1 | hybrid\_PKS/NRPS\_(PKSI) | BGC0000952\_c3 | Pristinamycin\_biosynthetic\_gene\_cluster | 32 | 24.8689852311 | 238 | 8.7e-62 |
| AHD05679.1 | putative\_non-ribosomal\_peptide\_ligase/\_polyketide\_synthase\_hybrid | BGC0000402 | Paenilarvins\_biosynthetic\_gene\_cluster | 31 | 25.0119104335 | 238 | 8.7e-62 |
| ADA69239.2 | trans-AT\_hybrid\_polyketide\_synthase-NRPS | BGC0001071 | Nosperin\_biosynthetic\_gene\_cluster | 29 | 24.7737017627 | 237 | 1.9e-61 |
| CBX87032.1 | BcBOA9\_protein | BGC0001243\_c3 | Botryenalol\_/\_botrydial\_/\_dihydrobotrydial\_/\_botryendial\_/ | 36 | 20.8670795617 | 236 | 2.5e-61 |
| AFX60341.1 | polyketide\_synthase | BGC0001032 | Oocydin\_A\_biosynthetic\_gene\_cluster | 31 | 24.8689852311 | 236 | 3.3e-61 |
| OAQ83765.1 | KR\_domain-containing\_protein | BGC0001358 | Leucinostatins\_biosynthetic\_gene\_cluster | 33 | 20.2953787518 | 235 | 7.4e-61 |
| CCM44330.1 | putative\_polyketide-type\_polyunsaturated\_fatty\_acid\_synthase | BGC0001056 | Zeamine\_biosynthetic\_gene\_cluster | 29 | 35.1119580753 | 234 | 1.3e-60 |
| EGJ35088.1 | polyketide\_synthase\_module | BGC0001163 | 1-heptadecene\_biosynthetic\_gene\_cluster | 28 | 27.3939971415 | 233 | 1.6e-60 |
| AAB81123.1 | unknown | BGC0000862 | Eicosapentaenoic\_acid\_biosynthetic\_gene\_cluster | 25 | 43.5445450214 | 233 | 2.2e-60 |
| CAG23961.2 | polyketide\_synthase | BGC0001089 | Bacillaene\_biosynthetic\_gene\_cluster | 31 | 25.6312529776 | 232 | 6.3e-60 |
| AEE88280.1 | CurJ | BGC0000976 | Curacin\_biosynthetic\_gene\_cluster | 28 | 27.1557884707 | 231 | 8.2e-60 |
| AAT70105.1 | CurJ | BGC0001165 | Curacin\_biosynthetic\_gene\_cluster | 28 | 27.1557884707 | 231 | 8.2e-60 |
| AKC54422.1 | fumosorinone\_biosynthesis\_polyketide\_synthase | BGC0001218 | Fumosorinone\_biosynthetic\_gene\_cluster | 34 | 21.6293473082 | 231 | 1.1e-59 |
| AAY89051.1 | hybrid\_nonribosomal\_peptide\_synthetase/polyketide\_synthase | BGC0001069 | Chivosazole\_biosynthetic\_gene\_cluster | 33 | 21.7246307766 | 229 | 4.1e-59 |
| ADN68482.1 | SorG | BGC0000184 | Sorangicin\_biosynthetic\_gene\_cluster | 31 | 25.6788947118 | 228 | 9e-59 |
| ABM63530.1 | BryD | BGC0000174 | Bryostatin\_biosynthetic\_gene\_cluster | 29 | 25.440686041 | 227 | 1.2e-58 |
| AHH34189.1 | polyketide\_synthase | BGC0001162 | 1-heptadecene\_biosynthetic\_gene\_cluster | 28 | 27.3939971415 | 227 | 2e-58 |
| ABC38101.1 | polyketide\_synthase | BGC0000964 | Burkholdac\_biosynthetic\_gene\_cluster | 32 | 24.4878513578 | 225 | 7.7e-58 |
| AHD05620.1 | putative\_polyketide\_synthase\_subunit | BGC0001033 | Paenilamicin\_biosynthetic\_gene\_cluster | 28 | 32.3010957599 | 225 | 7.7e-58 |
| AFR69332.1 | polyketide\_synthase\_SpiB | BGC0001045 | Spiruchostatin\_biosynthetic\_gene\_cluster | 30 | 26.4888041925 | 219 | 4.2e-56 |
| AFV52145.1 | polyketide\_synthase | BGC0000081 | Kedarcidin\_biosynthetic\_gene\_cluster | 26 | 42.7822772749 | 218 | 7.2e-56 |
| CBK62718.1 |  | BGC0001115 | Thiomarinol\_biosynthetic\_gene\_cluster | 27 | 30.9194854693 | 217 | 1.6e-55 |
| ABS74181.1 | BmyA | BGC0001090 | Bacillomycin\_biosynthetic\_gene\_cluster | 30 | 24.8213434969 | 217 | 2.1e-55 |
| BAB69698.1 | iturin\_A\_synthetase\_A | BGC0001098 | Iturin\_biosynthetic\_gene\_cluster | 30 | 24.8213434969 | 217 | 2.1e-55 |
| AEH59110.1 | polyketide\_synthase\_of\_type\_I | BGC0000385 | Lysobactin\_biosynthetic\_gene\_cluster | 31 | 25.0119104335 | 215 | 6.1e-55 |
| ADH01484.1 | putative\_type-I\_PKS | BGC0000995 | FR901464\_biosynthetic\_gene\_cluster | 29 | 25.9171033826 | 214 | 1e-54 |
| AIC32692.1 | FR9C | BGC0001113 | Spliceostatin\_/\_FR901464\_biosynthetic\_gene\_cluster | 29 | 25.9171033826 | 214 | 1e-54 |
| AAM78012.1 | warhead-forming\_iterative\_polyketide\_synthase | BGC0000112 | Neocarzinostatin\_biosynthetic\_gene\_cluster | 25 | 41.3530252501 | 214 | 1.4e-54 |
| ABS90478.1 | PKS | BGC0001106 | Oxazolomycin\_biosynthetic\_gene\_cluster | 29 | 28.0133396856 | 213 | 1.8e-54 |
| ABC34154.1 | thiotemplate\_mechanism\_natural\_product\_synthetase | BGC0000961 | Bactobolin\_biosynthetic\_gene\_cluster | 30 | 25.2024773702 | 211 | 8.8e-54 |
| AJO72734.1 | Type\_I\_modular\_polyketide\_synthase | BGC0001381 | Brasilinolide\_biosynthetic\_gene\_cluster | 32 | 22.0104811815 | 211 | 8.8e-54 |
| ACY06292.1 | modular\_polyketide\_synthase | BGC0001042 | Sanglifehrin\_A\_biosynthetic\_gene\_cluster | 30 | 30.0619342544 | 210 | 2.5e-53 |
| CAD15512.1 | probable\_polyketide\_synthase\_protein | BGC0001014 | Micacocidin\_biosynthetic\_gene\_cluster | 31 | 24.6784182944 | 209 | 4.3e-53 |
| CCP45170.1 | Polyketide\_synthetase\_MbtC\_(polyketide\_synthase) | BGC0001021 | Mycobactin\_biosynthetic\_gene\_cluster | 32 | 20.5335874226 | 208 | 9.7e-53 |
| AAQ17110.2 | enediyne\_polyketide\_synthase | BGC0001008 | Maduropeptin\_biosynthetic\_gene\_cluster | 26 | 35.7789423535 | 206 | 2.8e-52 |
| AGN11880.1 | TstC | BGC0001114 | Thailanstatin\_biosynthetic\_gene\_cluster | 31 | 21.4387803716 | 203 | 2.4e-51 |
| ACY01400.1 | AT-less\_polyketide\_synthase | BGC0000083 | Lactimidomycin\_biosynthetic\_gene\_cluster | 30 | 22.2963315865 | 202 | 4.1e-51 |
| BAD55609.1 | putative\_polyketide\_synthase | BGC0001027 | Nocobactin\_NA\_biosynthetic\_gene\_cluster | 31 | 20.5335874226 | 201 | 9.1e-51 |
| OAQ83760.1 | polyketide\_synthase | BGC0001358 | Leucinostatins\_biosynthetic\_gene\_cluster | 31 | 20.7241543592 | 199 | 3.4e-50 |
| ACI12948.1 | PfaA | BGC0000861 | Eicosapentaenoic\_acid\_biosynthetic\_gene\_cluster | 29 | 22.2486898523 | 191 | 9.4e-48 |
| AAM12934.1 | MmpF | BGC0000182 | Mupirocin\_biosynthetic\_gene\_cluster | 26 | 30.3954263935 | 186 | 3e-46 |
| CAJ76289.1 | putative\_hybrid\_non-ribosomal\_peptide-polyketide\_synthetase | BGC0000972 | Colibactin\_biosynthetic\_gene\_cluster | 28 | 25.0595521677 | 180 | 2.2e-44 |
| AGN74895.1 | nonribosomal\_peptide\_synthetase/polyketide\_synthase\_hybrid\_protein | BGC0000459 | Griseoviridin\_/\_viridogrisein\_biosynthetic\_gene\_cluster | 27 | 29.2520247737 | 169 | 5e-41 |
| BAF50720.1 | hybrid\_non\_ribosomal\_peptide\_synthetase-polyketide\_synthase | BGC0001116 | Virginiamycin\_biosynthetic\_gene\_cluster | 27 | 25.2024773702 | 167 | 1.9e-40 |
| CBW45740.1 | hybrid\_NRPS/PKS\_(PKSIV) | BGC0000952\_c3 | Pristinamycin\_biosynthetic\_gene\_cluster | 26 | 25.3454025727 | 166 | 4.2e-40 |
| AEP40937.1 | polyketide\_synthase\_type\_I | BGC0000021 | Apoptolidin\_biosynthetic\_gene\_cluster | 29 | 21.0100047642 | 163 | 2.7e-39 |
| ALD82524.1 | polyketide\_synthase | BGC0001212 | Nannocystin\_biosynthetic\_gene\_cluster | 26 | 28.5373987613 | 160 | 1.8e-38 |
| AGJ76603.1 | HglC | BGC0000869 | Heterocyst\_glycolipids\_biosynthetic\_gene\_cluster | 22 | 49.5950452596 | 159 | 5.2e-38 |
| WP\_048832936.1 | polyketide\_synthase | BGC0001348 | JBIR-100\_biosynthetic\_gene\_cluster | 27 | 21.7246307766 | 157 | 1.5e-37 |
| AAO39107.1 | AdmM | BGC0000956 | Andrimid\_biosynthetic\_gene\_cluster | 27 | 25.2024773702 | 157 | 2e-37 |
| AGI61676.1 | modular\_PKS | BGC0000095 | Micromonolactam\_biosynthetic\_gene\_cluster | 25 | 36.7794187708 | 150 | 2.4e-35 |
| BAB69199.1 | modular\_polyketide\_synthase | BGC0000117 | Oligomycin\_biosynthetic\_gene\_cluster | 26 | 26.1553120534 | 147 | 2e-34 |
| BAP34734.1 | type\_I\_polyketide\_synthase | BGC0000078 | Incednine\_biosynthetic\_gene\_cluster | 25 | 32.2534540257 | 144 | 1e-33 |
| AIA58899.1 | HRPKS | BGC0001141 | Brefeldin\_biosynthetic\_gene\_cluster | 25 | 21.7722725107 | 125 | 8.3e-28 |
| CAE51182.1 | RemE\_protein | BGC0000264 | Resistomycin\_biosynthetic\_gene\_cluster | 28 | 20.6288708909 | 124 | 1.1e-27 |
| CAE45667.1 | borrelidin\_polyketide\_synthase,\_type\_I | BGC0000031 | Borrelidin\_biosynthetic\_gene\_cluster | 24 | 20.1048118152 | 108 | 1e-22 |
| BAF85838.1 | modular\_polyketide\_synthase | BGC0000109 | Nemadectin\_biosynthetic\_gene\_cluster | 22 | 21.7722725107 | 100 | 2.8e-20 |
